# COVID-19 Rebound After VV116 vs Nirmatrelvir-Ritonavir Treatment: A Randomized Clinical Trial

**DOI:** 10.1001/jamanetworkopen.2024.1765

**Published:** 2024-03-13

**Authors:** Zhitao Yang, Yu Xu, Ruizhi Zheng, Lei Ye, Gang Lv, Zhujun Cao, Rulai Han, Mian Li, Yuanyue Zhu, Qiuyu Cao, Yi Ding, Jiqiu Wang, Yun Tan, Feng Liu, Dong Wei, Wei Tan, Weiwei Jiang, Jing Sun, Shouyue Sun, Jie Shao, Yang Deng, Weiyi Gao, Weiqing Wang, Ren Zhao, Liping Qiu, Erzhen Chen, Xinxin Zhang, Shengyue Wang, Guang Ning, Yiping Xu, Yufang Bi

**Affiliations:** 1 Emergency Department, Ruijin Hospital, Shanghai Jiao Tong University School of Medicine, Shanghai, China; 2 Department of Endocrine and Metabolic Diseases, Shanghai Institute of Endocrine and Metabolic Diseases, Ruijin Hospital, Shanghai Jiao Tong University School of Medicine, Shanghai, China; 3 National Clinical Research Center for Metabolic Diseases (Shanghai), Key Laboratory for Endocrine and Metabolic Diseases of the National Health Commission, National Research Center for Translational Medicine, State Key Laboratory of Medical Genomics, Ruijin Hospital, Shanghai Jiao Tong University School of Medicine, Shanghai, China; 4 Shanghai Institute of Hematology, National Research Center for Translational Medicine, State Key Laboratory of Medical Genomics, Ruijin Hospital, Shanghai Jiao Tong University School of Medicine, Shanghai, China; 5 Department of Infectious Diseases, Ruijin Hospital, Shanghai Jiao Tong University School of Medicine, Shanghai, China; 6 Department of Geriatrics, Medical Center on Aging, Ruijin Hospital, Shanghai Jiao Tong University School of Medicine, Shanghai, China; 7 Department of Infectious Diseases, Research Laboratory of Clinical Virology, National Research Center for Translational Medicine, Ruijin Hospital, Shanghai Jiao Tong University School of Medicine, Shanghai, China; 8 Research and Development Administration Department, Ruijin Hospital, Shanghai Jiao Tong University School of Medicine, Shanghai, China; 9 Department of Gastroenterology, Ruijin Hospital, Shanghai Jiao Tong University School of Medicine, Shanghai, China; 10 Department of Pediatrics, Ruijin Hospital, Shanghai Jiao Tong University School of Medicine, Shanghai, China; 11 Department of General Surgery, Ruijin Hospital, Shanghai Jiao Tong University School of Medicine, Shanghai, China; 12 Division of Medical Affairs, Ruijin Hospital, Shanghai Jiao Tong University School of Medicine, Shanghai, China; 13 Administrative Office, Ruijin Hospital, Shanghai Jiao Tong University School of Medicine, Shanghai, China; 14 Clinical Trials Center, Ruijin Hospital, Shanghai Jiao Tong University School of Medicine, Shanghai, China

## Abstract

**Importance:**

With the widespread use of anti–SARS-CoV-2 drugs, accumulating data have revealed potential viral load rebound after treatment.

**Objective:**

To compare COVID-19 rebound after a standard 5-day course of antiviral treatment with VV116 vs nirmatrelvir-ritonavir.

**Design, Setting, and Participants:**

This is a single-center, investigator-blinded, randomized clinical trial conducted in Shanghai, China. Adult patients with mild-to-moderate COVID-19 and within 5 days of SARS-CoV-2 infection were enrolled between December 20, 2022, and January 19, 2023, and randomly allocated to receive either VV116 or nirmatrelvir-ritonavir.

**Interventions:**

Participants in the VV116 treatment group received oral 600-mg VV116 tablets every 12 hours on day 1 and 300 mg every 12 hours on days 2 through 5. Participants in the nirmatrelvir-ritonavir treatment group received oral nirmatrelvir-ritonavir tablets with 300 mg of nirmatrelvir plus 100 mg of ritonavir every 12 hours for 5 days. Participants were followed up every other day until day 28 and every week until day 60.

**Main Outcomes and Measures:**

The primary outcome was viral load rebound (VLR), defined as a half-log increase in viral RNA copies per milliliter compared with treatment completion. Secondary outcomes included a reduction in the cycle threshold value of 1.5 or more, time until VLR, and symptom rebound, defined as an increase of more than 2 points in symptom score compared with treatment completion. The primary outcome and secondary outcomes were analyzed using the full analysis set. Sensitivity analyses were conducted using the per protocol set. Adverse events were analyzed using the safety analysis set.

**Results:**

The full analysis set included 345 participants (mean [SD] age, 53.2 [16.8] years; 175 [50.7%] were men) who received VV116 (n = 165) or nirmatrelvir-ritonavir (n = 180). Viral load rebound occurred in 33 patients (20.0%) in the VV116 group and 39 patients (21.7%) in the nirmatrelvir-ritonavir group (*P* = .70). Symptom rebound occurred in 41 of 160 patients (25.6%) in the VV116 group and 40 of 163 patients (24.5%) in the nirmatrelvir-ritonavir group (*P* = .82). Viral whole-genome sequencing of 24 rebound cases revealed the same lineage at baseline and at viral load rebound in each case.

**Conclusions and Relevance:**

In this randomized clinical trial of patients with mild-to-moderate COVID-19, viral load rebound and symptom rebound were both common after a standard 5-day course of treatment with either VV116 or nirmatrelvir-ritonavir. Prolongation of treatment duration might be investigated to reduce COVID-19 rebound.

**Trial Registration:**

Chinese Clinical Trial Registry Identifier: ChiCTR2200066811

## Key Points

**Question:**

How common is COVID-19 rebound after a standard 5-day course of treatment with VV116 vs nirmatrelvir-ritonavir?

**Findings:**

In this randomized clinical trial of 345 patients with mild-to-moderate COVID-19, viral load rebound occurred in 20.0% of patients in the VV116 group and 21.7% of patients in the nirmatrelvir-ritonavir group. Symptom rebound occurred in 25.6% of patients in the VV116 group and 24.5% of patients in the nirmatrelvir-ritonavir group.

**Meaning:**

Viral load rebound and symptom rebound are both common and not significantly different after a standard 5-day course of treatment with either VV116 or nirmatrelvir-ritonavir for mild-to-moderate COVID-19.

## Teaser

This randomized clinical trial compares COVID-19 rebound after a standard 5-day course of antiviral treatment with VV116 vs nirmatrelvir-ritonavir.

## Introduction

With the widespread use of anti–SARS-CoV-2 drugs, accumulating data revealed potential viral load rebound (VLR) after treatment. Cases of VLR in COVID-19 were first reported in early 2022 among patients receiving nirmatrelvir-ritonavir treatment, documenting a COVID-19 “recrudescence” featured by viral load fluctuation in parallel with typical symptoms.^1,2^ In May 2022, the US Centers for Disease Control and Prevention (CDC) issued an official health advisory on the potential for recurrence of COVID-19 or “COVID-19 rebound” after nirmatrelvir-ritonavir treatment.^3^

Afterward, information on COVID-19 rebound was soon reported in patient populations using clinical data. By retrospectively reviewing hospitalized patients’ records, a rebound rate between 0.8% and 6.6% was reported in different studies in patients with mild-to-moderate COVID-19 receiving nirmatrelvir-ritonavir, molnupiravir, or no treatment.^4,5,6,7^ However, these are retrospective studies, in which the incidence of disease rebound could have been underestimated due to the lack of a specifically designed and active monitoring schedule. In addition, the length and frequency of follow-up were usually not adequate. The definition of disease rebound was also very different between studies.

Meanwhile, new drugs have been developed to treat SARS-CoV-2 infection. Among them, VV116, a new orally available remdesivir derivative and RNA-dependent RNA polymerase inhibitor, was reported to be noninferior to nirmatrelvir-ritonavir in shortening the time to sustained clinical recovery with fewer adverse events among patients with mild-to-moderate COVID-19.^8^ To our knowledge, VLR after treatment with VV116 is unknown. Previous studies comparing rebound rates between antiviral drugs were based on observational data, which are inherently limited by unmeasured or unknown confounding. Therefore, we conducted a randomized clinical trial to measure and compare COVID-19 rebound after antiviral treatments with VV116 and nirmatrelvir-ritonavir by actively and frequently monitoring changes in viral load and clinical symptoms for approximately 60 days.

## Methods

### Participants

The present study is a single-center, investigator-blinded, randomized clinical trial comparing the incidence of VLR and symptom rebound after a 5-day treatment with VV116 or nirmatrelvir-ritonavir during 60 days of follow-up after randomization (Chinese Clinical Trial Registry Identifier: ChiCTR2200066811). Participants were recruited from both outpatient and inpatient departments of Ruijin Hospital, affiliated with Shanghai Jiao Tong University School of Medicine, and were enrolled between December 20, 2022, and January 19, 2023. Patients with mild-to-moderate COVID-19 who were 18 years of age or older and within 5 days of their first positive test result for SARS-CoV-2 infection were eligible to participate. Key exclusion criteria included high risk for severe COVID-19, previous anti–SARS-CoV-2 treatment, contraindications to study drugs or currently taking drugs contraindicated with nirmatrelvir tablets or ritonavir tablets, abnormal liver or kidney function, and other safety concerns. A complete list of eligibility criteria is provided in the trial protocol in Supplement 1. The study was approved by the Medical Ethics Committee of Ruijin Hospital. All study participants provided written informed consent before screening. The study followed the Consolidated Standards of Reporting Trials (CONSORT) reporting guideline.

### Randomization

Block randomization with a block size of 4 was used to randomize eligible participants in a 1:1 ratio to receive either VV116 or nirmatrelvir-ritonavir. Participants and study staff who were responsible for study drug distribution and recycling were not blinded to the treatment assignment. Study investigators, including study physicians who treated participants and collected data on medical history and symptoms, study nurses who collected oropharyngeal swab samples, laboratory technicians who tested for viral load, and statisticians who did data analysis, were blinded to the treatment assignment.

### Intervention and Follow-Up

Participants were given VV116 oral tablets or nirmatrelvir-ritonavir oral tablets after randomization according to their treatment allocation. They were instructed to take oral 600-mg VV116 tablets every 12 hours on day 1 and 300 mg every 12 hours on days 2 through 5 or oral nirmatrelvir-ritonavir tablets with 300 mg of nirmatrelvir plus 100 mg of ritonavir every 12 hours for 5 days. Participants were required to record their intake of the study drugs in a medication log.

Starting from day 6, after completion of the 5-day course of study drug treatment, participants were asked to return every other day until day 28 and then every week until day 60. At each visit, a trained study nurse collected oropharyngeal swab samples from each participant, and the cycle threshold (Ct) value on each visit day was tested using quantitative reverse transcription–polymerase chain reaction (RT-qPCR) for SARS-CoV-2. In addition, COVID-19–related target symptoms were evaluated on each follow-up day using an expanded 23-item symptoms scoring system.^9^ A 4-point Likert scale was used for each symptom reported, where 0 indicated absent, 1 indicated mild, 2 indicated moderate, and 3 indicated severe, by asking the participant “what was the severity of your [insert symptom] at its worst over the last 24 hours?” The range of the overall score is 0 to 69, with higher scores indicating greater symptom severity.

### Outcome and Safety Assessments

The primary outcome was the occurrence of VLR, defined as a half-log increase in viral RNA copy numbers per milliliter at any of the follow-up time points compared with treatment completion (day 6). Secondary outcomes included (1) a reduction in Ct value of 1.5 or more compared with treatment completion; (2) time until VLR from treatment completion; (3) the occurrence of VLR within 7, 14, 21, and 28 days after treatment completion; (4) the occurrence of symptom rebound, defined as an increase of more than 2 points in symptom score compared with treatment completion; and (5) the occurrence of sustained symptom rebound, defined as an increase of more than 2 points in symptom score on 2 consecutive follow-up visits compared with treatment completion. Adverse events leading to discontinuation of study drugs and serious adverse events (SAEs) were reported. The relation of adverse events and SAEs with the study intervention was judged by a study physician (Z.C.).

### Laboratory Measurements

With primers and probes targeting *ORF1ab* and *N* genes of SARS-CoV-2, viral load was measured using (1) the RT-qPCR reported in Ct values and (2) the droplet digital polymerase chain reaction (ddPCR) reported in copies per milliliter. Quantitative reverse transcription–polymerase chain reaction was conducted on each follow-up day using the SARS-CoV-2 RNA detection kit (Shanghai BioGerm Medical Technology Co Ltd) with the SLAN real-time PCR System. Samples were stored at −80 °C before measurement using ddPCR on the Sniper DQ24 Digital PCR System (Sniper Medical Technology Co) using the Sniper Novel Coronavirus (SARS-CoV-2) Nucleic Acid Detection Kit.

For participants with a Ct reduction of 1.5 or more during follow-up, we genotyped SARS-CoV-2 isolates from oropharyngeal swab samples collected at baseline (day 0) and on the day with Ct reduction (day X) for that participant, provided that both day 0 and day X Ct values were less than 35 to aim for good genotyping quality. A total of 32 sample pairs were sent for viral whole-genome sequencing at the National Research Center for Translational Medicine (eMethods in Supplement 2).

### Statistical Analysis

The sample size was determined by assuming a VLR rate of 8% in the nirmatrelvir-ritonavir group, a reduction in the VLR rate of 6% in the VV116 group, a 2-sided α level of .05, and a statistical power of 80%. A total of 478 participants were needed, with 239 participants in each group. The first participant was enrolled on December 20, 2022, during a wave of infection with the Omicron variant in Shanghai starting from early December.^10^ This wave of infection peaked at the end of December, followed by a rapid decline in January. A total of 418 patients were screened by January 19, 2023, among which 374 eligible patients were randomized (eFigure 1 in Supplement 2). Examination of cases of VLR based on Ct values revealed a much higher-than-anticipated rate in the overall study population (65 cases in 374 enrolled participants [17.4%] by January 27, 2023). The statistical power exceeded 80% based on the updated rebound rate and a sample size of 374; thus, a decision to stop screening patients was made.

Three data analysis sets, including the full analysis set, the per protocol set, and the safety analysis set, were defined in the trial protocol in Supplement 1. The primary outcome and secondary outcomes were analyzed using the full analysis set. Sensitivity analyses were conducted using the per protocol set. Adverse events were analyzed using the safety analysis set. We used the χ^2^ test to compare the VLR rate between treatment groups. If the conditions for the χ^2^ test were not met, the Fisher exact test was used. The Kaplan-Meier method was used to estimate the median time to VLR since treatment completion, with the 95% CI estimated by means of the Brookmeyer-Crowley method with log-log transformation. The hazard ratio for VLR and its 95% CI were estimated with the use of the Cox proportional hazards regression model. Data for participants without VLR were censored on the last day of visit. Subgroup analyses of the primary outcome were prespecified to assess the consistency of the treatment effect. Interactions between treatment effects and subgroups were assessed with the product terms. Post hoc analyses were conducted to examine characteristics associated with the VLR rates. Generalized additive mixed models were used to fit the trajectories of log-transformed viral RNA copy numbers per milliliters by groups with or without VLR. Additional details are provided in the statistical analysis plan in Supplement 1. All *P* values were from 2-sided tests and results were deemed statistically significant at *P* < .05.

## Results

### Participants

**Figure 1.**
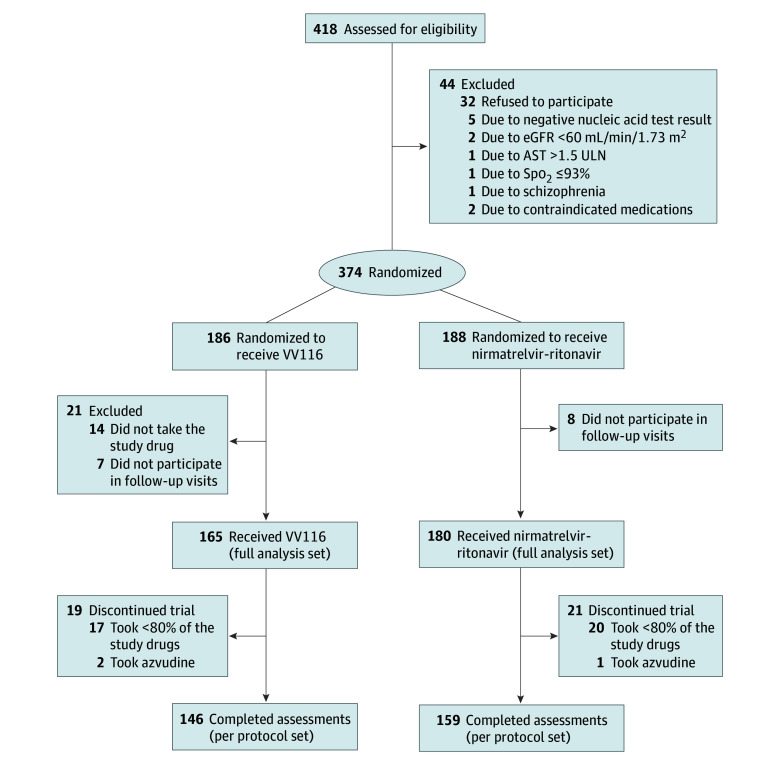
Flowchart of Study Participants AST indicates aspartate aminotransferase; eGFR, estimated glomerular filtration rate; Spo_2_, oxygen saturation; and ULN, upper limit of normal.

A total of 186 patients were assigned to receive VV116 and 188 patients were assigned to receive nirmatrelvir-ritonavir (Figure 1). Because 14 patients did not take the study drug and 15 patients did not participate in follow-up visits, 345 patients were included in the full analysis set. Overall, the mean (SD) age was 53.2 (16.8) years (range, 18-94 years), 117 participants (33.9%) were 60 years of age or older, 175 participants (50.7%) were men, and 170 participants (49.3%) were women (Table 1). More than 90% of participants (320 [92.8%]) were vaccinated. The baseline characteristics were balanced between the VV116 group (165 participants) and the nirmatrelvir-ritonavir group (180 participants). In addition, 37 participants took less than 80% of study drugs and 3 participants took azvudine; they were excluded from the per protocol set (Figure 1).

**Table 1.** Characteristics of the Study Participants in the Full Analysis Set

Characteristic	Total (N = 345)	VV116 (n = 165)	Nirmatrelvir-ritonavir (n = 180)
Age, mean (SD), y	53.2 (16.8)	52.8 (16.5)	53.8 (17.6)
Age group, No. (%)			
<60 y	228 (66.1)	106 (64.2)	122 (67.8)
≥60 y	117 (33.9)	59 (35.8)	58 (32.2)
Gender, No. (%)			
Men	175 (50.7)	82 (49.7)	93 (51.7)
Women	170 (49.3)	83 (50.3)	87 (48.3)
Vaccination status, No. (%)			
Unvaccinated	22 (6.4)	12 (7.3)	10 (5.6)
1-2 Doses	191 (55.4)	91 (55.1)	100 (55.6)
Booster dose	129 (37.4)	61 (37.0)	68 (37.8)
Missing	3 (0.9)	1 (0.6)	2 (1.1)
BMI, mean (SD)	24.0 (3.7)	24.0 (4.0)	23.9 (3.4)
History of disease, No. (%)			
Cardiovascular disease, including hypertension	102 (29.6)	37 (22.4)	65 (36.1)
Diabetes	36 (10.4)	14 (8.5)	22 (12.2)
Chronic lung disease	20 (5.8)	9 (5.5)	11 (6.1)
Chronic kidney disease	7 (2.0)	5 (3.0)	2 (1.1)
Score for COVID-19–related symptoms at randomization, median (IQR)	12 (8-19)	12 (8-18)	11 (8-19)
Time from first positive test result for SARS-CoV-2 to first dose of study drugs, median (IQR), d	1 (1-2)	1 (1-2)	1 (1-2)

Abbreviation: BMI, body mass index (calculated as weight in kilograms divided by height in meters squared).

### Outcomes

**Figure 2.**
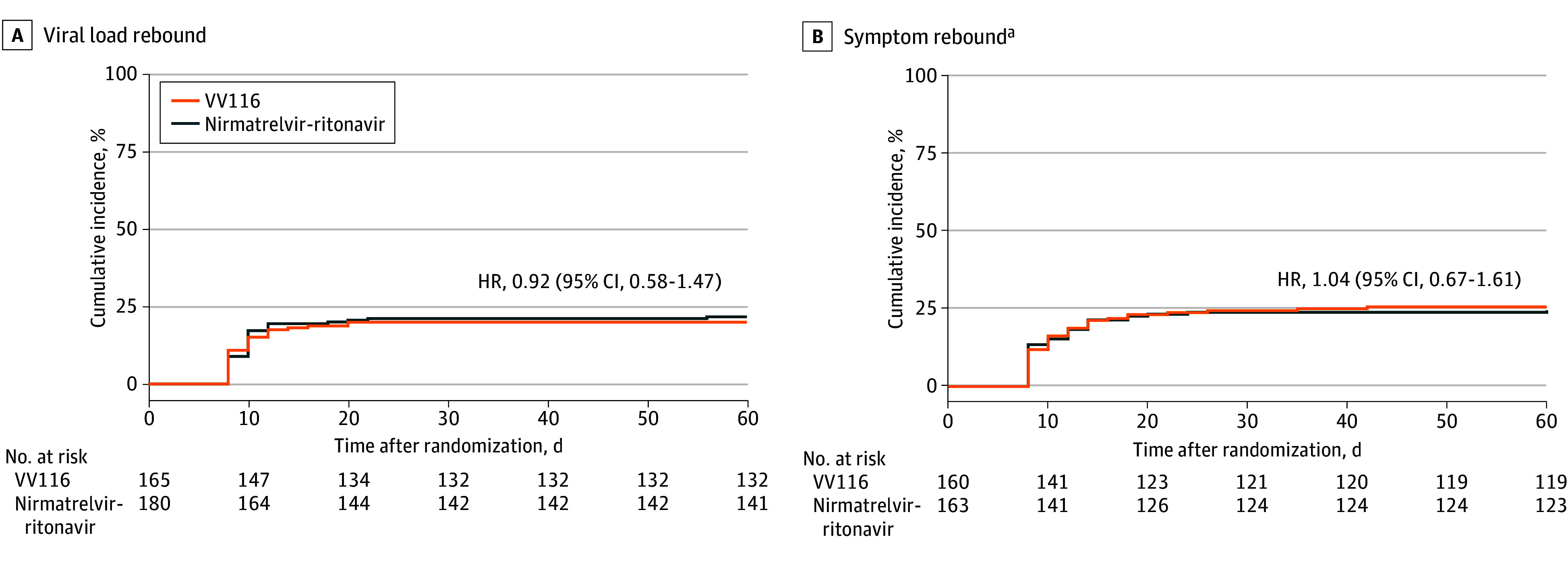
Occurrence of Viral Load Rebound and Symptom Rebound in Treatment Groups Receiving VV116 and Nirmatrelvir-Ritonavir in the Full Analysis Set ^a^Twenty-two participants were not included in the analysis of symptom rebound due to missing symptom scores on day 6.

Thirty-three participants (20.0%) in the VV116 group and 39 participants (21.7%) in the nirmatrelvir-ritonavir group had VLR, defined as a half-log increase in viral RNA copy numbers per milliliter compared with treatment completion (Table 2); there was no significant difference between the treatment groups in the rebound rate (*P* = .70). The occurrence of VLR over time is shown in Figure 2. Similar results were found in prespecified subgroups (Table 3) or using the per protocol set for analysis (eTable 1 in Supplement 2). Because all 14 participants who declined to take the assigned study drug were in the VV116 group, we performed a sensitivity analysis in which all 14 participants were assumed to have experienced VLR. The results showed that 47 participants (28.5%) in the VV116 group and 39 participants (21.7%) in the nirmatrelvir-ritonavir group had VLR (*P* = .14 for treatment group comparison). A total of 34 participants (20.6%) in the VV116 group and 33 participants (18.3%) in the nirmatrelvir-ritonavir group had a reduction in Ct value of 1.5 or more compared with treatment completion (*P* = .59). The median time to VLR since treatment completion was 8 days (95% CI, 8-10 days) in the VV116 group and 10 days (95% CI, 8-10 days) in the nirmatrelvir-ritonavir group (*P* = .47). There were 29 VLR cases (17.6%) in the VV116 group and 35 VLR cases (19.4%) in the nirmatrelvir-ritonavir group observed within 7 days after treatment completion (*P* = .66). Likewise, no significant differences were found between treatment groups in VLR rates within 14, 21, or 28 days after treatment completion.

**Table 2.** Primary and Secondary Outcomes in the Full Analysis Set

Outcome	VV116 (n = 165)	Nirmatrelvir-ritonavir (n = 180)	*P* value
Primary outcome			
Viral rebound, No. (%)	33 (20.0)	39 (21.7)	.70
Secondary outcomes			
Reduction in Ct value ≥1.5, No. (%)	34 (20.6)	33 (18.3)	.59
Time to VLR since treatment completion, median (95% CI), d	8 (8-10)	10 (8-10)	.47
VLR within 7 d after treatment completion, No. (%)	29 (17.6)	35 (19.4)	.66
VLR within 14 d after treatment completion, No. (%)	33 (20.0)	37 (20.6)	.90
VLR within 21 d after treatment completion, No. (%)	33 (20.0)	38 (21.1)	.80
VLR within 28 d after treatment completion, No. (%)	33 (20.0)	38 (21.1)	.80
Symptom rebound, No./total No. (%)^a^	41/160 (25.6)	40/163 (24.5)	.82
Sustained symptom rebound, No./total No. (%)^a^	19/160 (11.9)	21/163 (12.9)	.78

Abbreviations: Ct, cycle threshold; VLR, viral load rebound.

^a^ Twenty-two participants were not included in the analysis due to missing symptom scores on day 6.

**Table 3.** Subgroup Analysis for the Primary Outcome in the Full Analysis Set

Subgroups	Participants who experienced COVID-19 rebound, No./total No.		*P* value	*P* value for interaction
	VV116	Nirmatrelvir-ritonavir		
Age categories				
<40 y	5/42 (11.9)	7/46 (15.2)	.65	.59
40-59 y	14/64 (21.9)	13/76 (17.1)	.48	
≥60 y	14/59 (23.7)	19/58 (32.8)	.28	
Gender				
Men	21/82 (25.6)	20/93 (21.5)	.52	.18
Women	12/83 (14.5)	19/87 (21.8)	.21	
BMI^a^				
<25	23/106 (21.7)	23/113 (20.4)	.81	.32
≥25	8/51 (15.7)	14/59 (23.7)	.29	
History of diabetes				
With	2/14 (14.3)	9/22 (40.9)	.14^b^	.10
Without	31/151 (20.5)	30/158 (19.0)	.73	
History of cardiovascular disease, including hypertension				
With	4/37 (10.8)	17/65 (26.2)	.07	.11
Without	29/128 (22.7)	22/115 (19.1)	.50	
Vaccination status^a^				
Unvaccinated	2/12 (16.7)	1/10 (10.0)	>.99^b^	.27
1-2 Doses	16/91 (17.6)	26/100 (26.0)	.16	
Booster dose	15/61 (24.6)	12/68 (17.6)	.33	
Time from first positive test result for SARS-CoV-2 to first dose of study drugs				
<3 d	29/134 (21.6)	35/146 (24.0)	.64	.77
≥3 d	4/31 (12.9)	4/34 (11.8)	>.99^b^	

Abbreviation: BMI, body mass index (calculated as weight in kilograms divided by height in meters squared).

^a^ Missing data from 16 participants for BMI and from 3 participants for vaccination status.

^b^ Fisher exact test was used.

The median score for COVID-19–related target symptoms decreased rapidly during and after treatment in both groups (eFigure 2 in Supplement 2). Symptom rebound occurred in 41 of 160 participants (25.6%) in the VV116 group and in 40 of 163 participants (24.5%) in the nirmatrelvir-ritonavir group (*P* = .82) (Table 2). Sustained symptom rebound occurred in 19 of 160 participants (11.9%) in the VV116 group and in 21 of 163 participants (12.9%) in the nirmatrelvir-ritonavir group (*P* = .78). We considered changing the threshold of symptom rebound to more than 7 points (approximately 10% of the maximum score of 69). The results showed that 13 of 160 participants (8.1%) in the VV116 group and 14 of 163 participants (8.6%) in the nirmatrelvir-ritonavir group had symptom rebound (*P* = .88). In addition, using a definition of an increase of more than 2 points in symptom score compared with preceding time points after treatment completion, 78 participants (47.3%) in the VV116 group and 81 participants (45.0%) in the nirmatrelvir-ritonavir group had symptom rebound (*P* = .67).

Characteristics that might be associated with the risk of VLR were examined in a post hoc analysis. Participants 60 years of age or older had a significantly increased 164% risk of VLR compared with participants younger than 40 years (odds ratio, 2.64; 95% CI, 1.21-5.75) (eTable 2 in Supplement 2). In addition, VLR was more common, although not significantly different, among patients who received the first dose of study drug within 3 days (64 of 280 [22.9%]) compared with 3 or more days (8 of 65 [12.3%]) after the first positive test result for SARS-CoV-2 (*P* = .06). Characteristics associated with VLR rate in the VV116 group and the nirmatrelvir-ritonavir group are presented in eTables 3 and 4, respectively, in Supplement 2. The viral load in log-transferred viral RNA copy numbers per milliliter in the groups of patients with and without VLR is presented in eFigure 3 in Supplement 2.

### Safety

A total of 353 participants were included in the safety analysis set (166 in the VV116 group and 187 in the nirmatrelvir-ritonavir group). Eight participants (2 in the VV116 group and 6 in the nirmatrelvir-ritonavir group) discontinued their study drug due to adverse effects, such as nausea, vomiting, diarrhea, and discomfort in the stomach (eTable 5 in Supplement 2). A total of 10 participants (4 in the VV116 group and 6 in the nirmatrelvir-ritonavir group) experienced hospitalizations for SAEs during the study period. One participant in the VV116 group died during the study period. This patient was admitted to the inpatient department due to severe COVID-19 shortly after recruitment and before starting intervention treatment. None of the SAEs was considered by the investigator to be related to the study treatment. Four participants with SAEs also had VLR. However, none of the SAEs occurred due to VLR because they took place before rebound onset.

### Viral Genome Typing

Among the 32 pairs sent for viral whole-genome sequencing, genotyping data of 24 pairs (8 in the VV116 group and 16 in the nirmatrelvir-ritonavir group) had a good or mediocre quality control status evaluated by Nextclade quality metrics and were included for analysis. There were 14 patients infected by the BA.5.2 lineage and 10 patients infected by the BF.7 lineage at baseline. The same lineage (BA.5.2 or BF.7) at baseline and at VLR was found in each sequenced patient case (eFigure 4 and eTable 6 in Supplement 2). In addition, no known drug resistance mutations were found in the *NSP5* gene (encoding the main protease of SARS-CoV-2, the molecular target of nirmatrelvir-ritonavir) in the nirmatrelvir-ritonavir group, or in the *NSP12* gene (encoding RNA-dependent RNA polymerase of SARS-CoV-2, the molecular target of VV116) in the VV116 group.

## Discussion

Our results demonstrated that VLR and symptom rebound are common in nonhospitalized patients with COVID-19 treated with VV116 or nirmatrelvir-ritonavir for 5 days, with rates of 20.0% and 21.7%, respectively, for VLR and 25.6% and 24.5%, respectively, for symptom rebound. Despite the high rebound rates, no patients had disease progression due to rebound. This study adds important evidence of COVID-19 rebound after antiviral treatment in mostly vaccinated patients with COVID-19 who were infected with Omicron variants.

After several case reports and a US CDC official health advisory on COVID-19 rebound after nirmatrelvir-ritonavir treatment,^1,2,3^ clinical studies have reported rebounds of symptoms, virologic burden, and hospitalizations among patients with or without antiviral treatment.^4,5,6^ In the post hoc analysis of the Evaluation of Protease Inhibition for COVID-19 in High-Risk Patients (EPIC-HR) trial, a similar incidence of VLR was observed in the nirmatrelvir-ritonavir group and the placebo group.^7^ The EPIC-HR trial was conducted among unvaccinated patents with COVID-19 who were infected mainly with the B.1.617.2 (Delta) variant.^11^ The current rebound cases are observed in a highly vaccinated population infected with the BA.5.2 or BF.7 (Omicron) variant.^10^ The incidence of VLR in our study after nirmatrelvir-ritonavir treatment was significantly higher than the rates reported in the EPIC-HR trial and other studies.^4,5,6,7^ This difference is probably due to the different viral variants and vaccination status, but the active and rigorous monitoring schedule used in our trial might play the most important part. Similar and high rebound rates were found in another study with rigorous monitoring of viral load and symptoms in 563 patients with mild-to-moderate COVID-19 receiving placebo (ACTIV-2/A5401 study).^12^ Viral load rebound was detected in 31% of participants and symptom rebound was identified in 26% of participants. However, that study was conducted in a largely unvaccinated population infected with pre-Omicron variants without antiviral treatment. Similar rebound rates were found in 2 other observational studies, showing a 20.8% and 27% occurrence, respectively, of VLR in the participants treated with nirmatrelvir-ritonavir.^13,14^

To our knowledge, there is currently no consensus definition of COVID-19 rebound across studies. Using a definition of a half-log increase from the nadir viral load after treatment completion, 37 participants (22.4%) in the VV116 group and 42 participants (23.3%) in the nirmatrelvir-ritonavir group had VLR (*P* = .84). Alternative definitions of symptom rebound, such as an increase of more than 2 points in symptom score compared with preceding time points after treatment completion or changing the threshold of symptom rebound to more than 7 points, revealed no significant difference between treatment groups. Therefore, using alternative definitions for COVID-19 rebound did not change the conclusions.

Meanwhile, VLR did not necessarily coincide with symptom rebound in our study (eFigure 4 in Supplement 2). Among 72 participants with VLR and 81 participants with symptom rebound, 21 participants had both VLR and symptom rebound and only 9 participants had them on the same visit day. Inconsistencies were also found in previous studies. Although symptom rebound and VLR, counted individually, were both high in the ACTIV-2/A5401 study in which patients received placebo, the combination of symptom rebound and VLR was observed in only 3% of participants.^12^ Another observational study in the US found that individuals who completed nirmatrelvir-ritonavir treatment and experienced symptom rebound were more likely to have VLR than those without symptom rebound.^13^ Symptom rebound could have several causes. One is viral dissemination into different anatomic compartments over time, and another is coinfection with another respiratory virus.^15^ Viral and symptom rebound might be distinct features that can occur in patients with mild-to-moderate COVID-19.

VV116 is a new orally administered COVID-19 drug, which inhibits viral replication by inducing mutagenesis during viral RNA synthesis.^16^ All 14 patients who declined to take the assigned study drug were in the VV116 group. However, even if we assumed that all patients who declined to take VV116 had VLR, the rebound rates were not different between the treatment groups. In addition, no significant differences were found in the increase in viral load from nadir to peak, viral load peak level, and duration of rebound between the VV116 group and the nirmatrelvir-ritonavir group. These results indicate that rebound was not unique to nirmatrelvir-ritonavir and may be associated with a persistent viral infection in the patients treated with either nirmatrelvir-ritonavir or VV116. This finding was proved by viral full-genome sequencing showing the same lineage in sample pairs at baseline and at rebound. Similar rebound rates after nirmatrelvir-ritonavir and molnupiravir treatment, or no treatment, were also found in previous studies,^5,6,7^ although not evaluated by randomization. Therefore, it is speculated that VLR could occur in patients with inadequate viral clearance.^5^ An insufficient drug exposure or insufficient duration of drug treatment is likely to result in incomplete viral clearance and, therefore, contribute to COVID-19 rebound, especially in participants with potentially reduced innate immune responses such as older patients, which was demonstrated in the post hoc analysis of the current trial.

### Limitations

This study has some limitations. First, the trial design originally planned for 478 patients to have an 80% power to detect an assumed lower VLR rate in the VV116 group than in the nirmatrelvir-ritonavir group. However, because new cases of SARS-CoV-2 infections stopped appearing in many communities after 4 to 6 weeks of the beginning of the Omicron variant outbreak,^11^ we were able to enroll only 374 patients. However, almost the same rebound rates between treatment groups suggest that, even if enough patients had been enrolled, the results would not have changed. Second, our observations could be affected by the underlying study population because the study enrolled largely vaccinated nonhospitalized patients infected with Omicron variants. Recent studies have suggested that neither vaccination nor infection with Omicron variants substantially change viral decay kinetics.^17,18^ Third, the study did not measure host immune responses. The presence of any host immunosuppression may lead to VLR and an immune response reacting to the sudden reappearance of viral antigen could be important in symptomatic rebound.^19^ Fourth, the study was not designed as a noninferiority trial. Therefore, no definitive claims can be made regarding the relative rate of VLR in patients receiving the different treatments.

## Conclusions

In this randomized clinical trial of COVID-19 rebound after antiviral treatment, VLR and symptom rebound were both common and not statistically different after a standard 5-day course of treatment with either VV116 or nirmatrelvir-ritonavir in patients with mild-to-moderate COVID-19 who were infected with Omicron variants. Effective treatment options in addition to the drug per se, especially the potential prolongation of treatment duration, as well as the interplay of viral infection, host immunity, and antiviral treatment in the process of rebound, warrant investigation in future studies.

## Notes

Supplement 1.
Trial Protocol and Statistical Analysis Plan

Supplement 2. **eMethods.** Genome Sequencing Procedures
eReferences.
**eTable 1.** Primary and Secondary Outcomes in the Per Protocol Set **eTable 2.** Post-Hoc Analysis of Characteristics Associated With Viral Rebound Rate **eTable 3.** Post-Hoc Analysis of Characteristics Associated With Viral Rebound Rate in VV116 Group **eTable 4.** Post-Hoc Analysis of Characteristics Associated With Viral Rebound Rate in Nirmatrelvir-Ritonavir Group **eTable 5.** Adverse Events in the Safety Analysis Set **eTable 6.** Whole-Genome Sequencing of SARS-CoV-2 in 24 Cases **eFigure 1.** Enrollment of Study Participants **eFigure 2.** Median (Interquartile Range) Score for COVID-19–Related Target Symptoms in Patients Receiving VV116 or Nirmatrelvir-Ritonavir by Follow-Up Days **eFigure 3.** Trajectories of Viral Copy Numbers (log10-Transformed) by Groups of Rebound and Not Rebound **eFigure 4.** SARS-CoV-2 Variants, Ct Values, and Symptom Scores in 24 Cases

Supplement 3.
Data Sharing Statement
