# Supplementary material for: COVID-19 Rebound After VV116 vs Nirmatrelvir-Ritonavir Treatment: A Randomized Clinical Trial
Source: JAMA Netw Open. 2024 Mar 13;7(3):e241765. doi: 10.1001/jamanetworkopen.2024.1765 (PMC10938176; doi:10.1001/jamanetworkopen.2024.1765)
Supplement: Supplement 1. — Trial Protocol and Statistical Analysis Plan [file jamanetwopen-e241765-s001.pdf]

Ruijin Hospital, Shanghai Jiaotong University School of Medicine

Research Project Protocol Involving Human Subjects

Study Title：	COVID-19 rebound after JT001 (VV116) and nirmatrelvir–ritonavir treatment in patients with mild to moderate COVID-19: A single-blind, parallel-group, randomized clinical trial
Principal Investigator：	Guang Ning, Yufang Bi, Yiping Xu
Study Start：	December 2022 ~

Ruijin Hospital, Shanghai Jiaotong University School of Medicine

December 24th, 2022

Version Number: 1.2

1. Study Summary

1.1 Synopsis

**Study Title:**	COVID-19 rebound after JT001 (VV116) and nirmatrelvir–ritonavir treatment in patients with mild to moderate COVID-19: A single-blind, parallel-group, randomized clinical trial
**Introduction:**	The study is designed to enroll a total of 478 mild to moderate COVID-19 patients from Ruijin Hosptial, who are randomly assigned to receive either JT001 (VV116) treatment or nirmatrelvir–ritonavir treatment for a duration of 5 days. The study aims to compare the incidence of viral rebound within 60 days after randomization.
**Objective:**	Compare the viral load rebound rate in mild to moderate COVID-19 patients after receiving VV116 or nirmatrelvir–ritonavir treatment.
**Study population:**	478 mild to moderate COVID-19 patients, aged 18 years and above, with positive SARS-CoV-2 test results within 5 days prior to randomization. Please refer to the inclusion criteria for details.
**Institution/Location:**	Ruijin Hospital, Shanghai Jiaotong University School of Medicine
**Study intervention:**	JT001 (VV116) treatment group: Study participants receive VV116 600 mg, Q12H treatment on the first day; VV116 300 mg, Q12H treatment from day 2 to day 5.<br>Nirmatrelvir–ritonavir treatment group: participants receive nirmatrelvir 300 mg + ritonavir 100 mg, Q12H treatment from day 1 to day 5.
**Study duration:**	The treatment intervention lasts 5 days. The follow-up period for monitoring the viral rebound rate extends to 60 days after randomization.
**Duration of subject participation:**	60 days

1.2 Study Flowchart

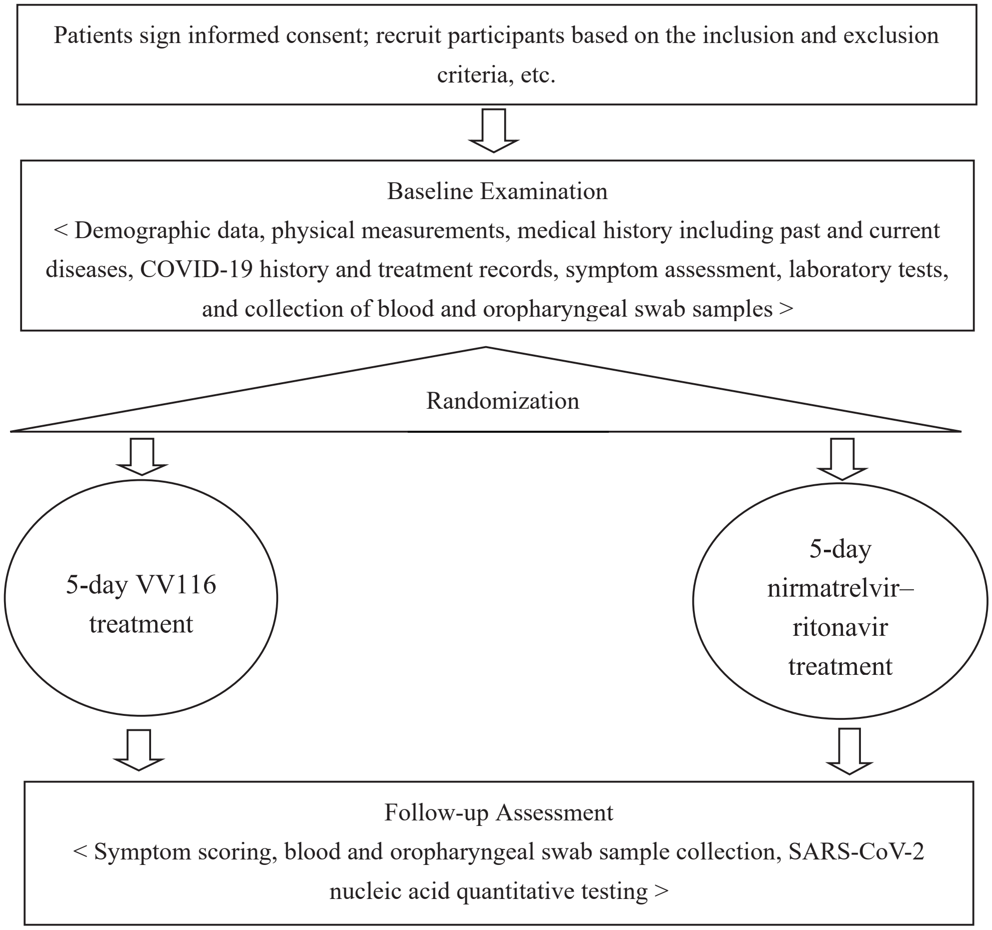

2. Background

With the widespread use of antiviral drugs for COVID-19, there have been reports of cases where the virus rebounds after completing the standard course of treatment. However, there is a lack of post-treatment nucleic acid monitoring data with a large sample size, and there may be differences in viral rebound among different drugs used in the treatment.

Nirmatrelvir–ritonavir is the recommended treatment drug for mild-to-moderate COVID-19 patients with a high risk of disease progression [1]. It has received Emergency Use Authorization (EUA) from the FDA and conditional approval from the National Medical Products Administration in China. In the Phase III clinical trial that included 2,246 patients, which was completed before the Omicron epidemic, the results showed that nirmatrelvir–ritonavir could reduce the risk of hospitalization or death by 89% [2]. Real-world data analysis of over one million community-infected non-hospitalized patients during the Omicron outbreak in Hong Kong in 2022 found that the early use of nirmatrelvir–ritonavir could significantly reduce the disease progression, hospitalization rates, and mortality compared to the control group without receiving antiviral treatment, confirming its effectiveness in the population infected with Omicron [3].

With the widespread use of nirmatrelvir–ritonavir, there have been reports of viral rebound after completing the treatment [4,5]. Although a post-hoc analysis of the EPIC-HR trial data found that the viral load rebound rate in the nirmatrelvir–ritonavir treatment group (2.3%) was not significantly higher than that of the control group (1.7%) [6], it is important to note that the study had limitations such as a small number of observations and a short observational period, which may have led to a significant underestimation of the viral load rebound rate. In a clinical trial with rigorous nucleic acid monitoring, among 568 patients in the placebo control group who underwent an average of 16 nucleic acid tests, the viral load rebound rate was 12% [7]. A meta-analysis of 20 studies found that the viral rebound rate within 14 days and beyond 14 days of follow-up was 11.5% and 14.5%, respectively [8]. These findings suggest that the estimation of the viral rebound rate is closely related to the frequency of observations and the duration of follow-up, and targeted trial designs are needed for evaluation.

In addition, the viral load rebound rate may vary between different treatments with antiviral drugs. Nirmatrelvir–ritonavir is a 3CL protease inhibitor for SARS-CoV-2, while other drugs with different mechanisms such as RNA-dependent RNA polymerase (RdRp) inhibitors, including Remdesivir, Molnupiravir, and VV116, may have different viral load rebound rates after treatment. Real-world studies conducted in Hong Kong and the United States have compared the viral load rebound rate after

treatment with nirmatrelvir–ritonavir and Molnupiravir, but no significant differences were found [9,10]. However, these studies with retrospective design lacked targeted evaluations of viral load rebound rates, thus requiring rigorous prospective clinical trials for further evaluation.

This study aims to compare the differences in viral load rebound rates between VV116 and nirmatrelvir–ritonavir after treatment through a single-blind, randomized controlled trial design.

References:

1. Agarwal A, Rochwerg B, Lamontagne F, et al. A living WHO guideline on drugs for covid-19. The BMJ 2020;370:m3379.

2. Hammond J, Leister-Tebbe H, Gardner A, et al. Oral Nirmatrelvir for High-Risk, Nonhospitalized Adults with Covid-19. N Engl J Med 2022;386:1397-408.

3. Wong CKH, Au ICH, Lau KTK, et al. Real-world effectiveness of molnupiravir and nirmatrelvir plus ritonavir against mortality, hospitalisation, and in-hospital outcomes among community-dwelling, ambulatory patients with confirmed SARS-CoV-2 infection during the omicron wave in Hong Kong: an observational study. Lancet. 2022 Oct 8;400(10359):1213-1222.

4. Callaway E. COVID rebound is surprisingly common - even without Paxlovid. Nature. 2022 Aug 11. doi: 10.1038/d41586-022-02121-z.

5. Charness ME, Gupta K, Stack G, Strymish J, Adams E, Lindy DC, Mohri H, Ho DD. Rebound of SARS-CoV-2 Infection after Nirmatrelvir-Ritonavir Treatment. N Engl J Med. 2022 Sep 15;387(11):1045-1047.

6. Anderson AS, Caubel P, Rusnak JM; EPIC-HR Trial Investigators. Nirmatrelvir-Ritonavir and Viral Load Rebound in Covid-19. N Engl J Med. 2022 Sep 15;387(11):1047-1049.

7. Deo R, Choudhary MC, Moser C, Ritz J, Daar ES, Wohl DA, Greninger AL, Eron JJ, Currier JS, Hughes MD, Smith DM, Chew KW, Li JZ; ACTIV-2/A5401 Study Team. Viral and Symptom Rebound in Untreated COVID-19 Infection. medRxiv [Preprint]. 2022 Aug 2:2022.08.01.22278278.

8. 张奕,孙瑛,段玮,等. 新型冠状病毒肺炎患者出院后核酸复阳发生率的 Meta 分析[J]. 国际病毒学杂志,2021,28(1):6-10. DOI:10.3760/cma.j.issn.1673-4092.2021.01.002.

9. Wong GL, Yip TC, Lai MS, Wong VW, Hui DS, Lui GC. Incidence of Viral Rebound After Treatment With Nirmatrelvir-Ritonavir and Molnupiravir. JAMA Netw Open. 2022 Dec 1;5(12):e2245086.

10. Wang L, Berger NA, Davis PB, Kaelber DC, Volkow ND, Xu R. COVID-19 rebound after Paxlovid and Molnupiravir during January-June 2022. medRxiv [Preprint]. 2022 Jun 22:2022.06.21.22276724. doi: 10.1101/2022.06.21.22276724. PMID: 35794889; PMCID: PMC9258292.

3. Objectives

- To determine the viral load rebound rate of SARS-CoV-2 in mild to moderate COVID-19 patients treated with VV116 compared to nirmatrelvir–ritonavir.
- To identify whether there is a significant difference in the viral load rebound rate in mild to moderate COVID-19 patients treated with VV116 compared to nirmatrelvir–ritonavir.

4. Risk/Benefit Assessment

4.1 Potential Risk

Overall, both treatment drugs have good safety and tolerability. Common adverse reactions of VV116 include abnormal laboratory tests (such as detection of urinary crystals, urinary red blood cells, leukocytes, bacteria, positive urinary sugar, decreased neutrophils, decreased platelets, elevated transaminases, elevated uric acid, and elevated triglycerides, etc.), gastrointestinal reactions (such as dry mouth, nausea, and bloating, etc.), abnormal electrocardiogram findings (such as shortened PR interval, first-degree atrioventricular block, and sinus bradycardia), and increased blood pressure. Common adverse reactions of nirmatrelvir–ritonavir include diarrhea and taste distortion, and rare adverse reactions include dyspepsia, gastroesophageal reflux disease, vomiting, myalgia, dizziness, elevated alanine transaminase, and elevated aspartate transaminase.

During the research process, conducting certain procedures may pose risks and discomfort. Here are some potential discomforts and corresponding measures for specific procedures:

- Venous blood collection: The study involves collecting blood samples from patients through the veins. Venous blood collection may cause discomfort and bruising at the site of the needle puncture, and there is a potential risk of infection. Other extremely rare risks include nerve damage, dizziness, and fainting.

- Oropharyngeal swab collection: Oropharyngeal swab collection is necessary for SARS-CoV-2 nucleic acid testing. Collecting a oropharyngeal swab may cause some discomfort to patients, and in extreme cases, there may be some minor injuries. However, this test is generally considered safe with minimal risks.

- Pregnant patients or those planning to become pregnant are excluded from the study. If a patient becomes pregnant during the research period, it may pose unknown risks to both the patient and the unborn fetus.

4.2 Known Potential Benefits

In vitro studies have demonstrated that VV116 can inhibit the replication of SARS-CoV-2 in a laboratory setting. This suggests that VV116 could potentially be an effective treatment for mild-to-moderate COVID-19 patients. Preclinical efficacy results from in vivo animal models also support the potential benefits of early treatment with VV116 for mild-to-moderate COVID-19 patients. Furthermore, results from a multicenter randomized controlled clinical trial conducted in China indicate that the clinical recovery time with VV116 treatment is non-inferior to the currently recommended first-line treatment drug, nirmatrelvir–ritonavir, and the safety is relatively good.

4.3 Potential Risk/Benefits Assessment

VV116 may or may not reduce the risk of viral load rebound after patient's testing result turns negative. However, the findings of this study can be used to assess the viral load rebound rates and differences in patients with mild-to-moderate COVID-19 treated with VV116 and nirmatrelvir–ritonavir, guiding clinical treatment and helping COVID-19 patients receive more effective treatment options and medications. Therefore, the study participants may potentially benefit from this study.

5. Information of Principal Investigators

Guang Ning, Chief physician, President of Ruijin Hospital

Yufang Bi, Chief physician, Vice President of Ruijin Hospital

Yiping Xu, Head of the Clinical Trials Center of Ruijin Hospital

6. Study Design

6.1 Overall design

This study is a single-center, single-blind, parallel-group, randomized controlled clinical trial. The study will screen and enroll 478 patients with mild-to-moderate COVID-19 who met the inclusion and exclusion criteria. These patients will be randomly assigned to receive either VV116 treatment or nirmatrelvir–ritonavir treatment for 5 days. After completing the treatment, participants will be followed up and undergo nucleic acid testing for 60 days to compare the viral load rebound after completing standard treatment between the two groups. The aim is to determine

whether VV116 treatment reduces the viral load rebound rate compared to nirmatrelvir–ritonavir treatment.

The study physicians will be blinded. The study will involve repeated nucleic acid testing in two groups of patients. The investigators responsible for sampling, testing, and evaluation will remain blinded to the study assignment.

The study will follow the intention-to-treat (ITT) principle and analysis of the data will be based on the randomization of study participants. A Chi-square test will be used to compare the viral rebound rate within 60 days after randomization.

6.2 Study Population

6.2.1 Inclusion Criteria

Participants are eligible to be enrolled in the study if all the following criteria apply:

1) Age ≥18 years at study entry;

2) ≤5 days after first positive test of SARS-CoV-2 virus infection on quantitative reverse-transcription polymerase chain reaction (RT-qPCR) or rapid antigen test;

3) Written informed consent.

6.2.2 Exclusion Criteria

Participants are excluded from the study if any of the following criteria apply:

1) Participants who are judged by the investigator as likely to progress to severe/critical COVID-19 prior to randomization;

2) Participants who have SpO_2 ≤93% on room air at sea level or PaO_2/FiO_2 ≤300, or respiratory rate ≥30 per minute;

3) Participants who require mechanical ventilation or anticipated impending need for mechanical ventilation;

4) Participants who have eye disease (such as inflammation, vessel deformity, retinal hemorrhage or decollement, optic nerve lesion, or fundus lesion);

5) Participants who have ALT or AST levels more than 1.5 times the upper limit of the normal range or eGFR <60 mL/min/1.73 m^2 within the past 30 days;

6) Participants who have known allergies to any of the components used in the formulation of the intervention drugs;

7) Participants with any medical condition, which judged by the investigator, will compromise the safety of the participant;

8) Participants who have received a SARS-CoV-2 monoclonal antibody treatment or prevention, or antiviral treatment (including the intervention drugs);

9) Participants who have received convalescent COVID-19 plasma treatment;

10) Participants who are taking contraindicated drugs in the Package Insert of nirmatrelvir tablets/ritonavir tablets (co-packaged);

11) Participants who have participated, within the last 30 days, in a clinical study involving an investigational intervention. If the previous investigational intervention has a long half-life, 5 half-lives or 30 days, whichever is longer, should have passed;

12) Participants who are concurrently enrolled in any other type of medical research judged not to be scientifically or medically compatible with this study;

13) Female participants who are pregnant or breast-feeding or plan to be pregnant within the study period;

14) Male participants whose wives or partners plan to be pregnant within the study period.

6.3 Participant Recruitment

Potential participants for this study will be recruited from the outpatient and inpatient departments of Ruijin Hospital affiliated with Shanghai Jiaotong University School of Medicine. The recruitment advertisement will be put on major social media channels such as WeChat and community residents living within a short radium of the hospital will be contacted by community workers for their inclination of participation. Potential participants are contacted by study staff and a screening visit is arranged, during which the written informed consent is obtained, and a detailed inquisition of medical history, physical examination, biochemical evaluation, and SARS-CoV-2 RT-PCR test is conducted to assess eligibility according to study inclusion and exclusion criteria.

6.4 Treatment Assignment

Block randomization with a block size of 4 is used to randomize eligible participants to receive either the VV116 treatment or the nirmatrelvir-ritonavir treatment in a 1:1 ratio. Randomization will be conducted at the Research Coordinating Center. After the study center screens and confirms eligibility of the participants according to the inclusion and exclusion criteria, the central randomization system will assign a random group number to each participant.

In this study, patients receiving drug treatment are not blinded to their treatment assignment. However, the research physicians, personnel responsible for sample collection, testing, and interpretation, as well as the statisticians are blinded to participants' treatment assignment.

6.5 Study Intervention

Participants who meet the inclusion criteria will be randomly assigned to one of the following groups:

A. VV116 treatment group: Participants will receive VV116 600 mg Q12H on day 1; VV116 300 mg Q12H on days 2-5.

B. Nirmatrelvir–ritonavir treatment group: Participants will receive nirmatavir 300 mg + ritonavir 100 mg Q12H on days 1-5.

Table. Dosage and administration method

	**JT001 (VV116)**	**nirmatrelvir tablet/ritonavir tablet (Paxloid)**
**Type**	Drug	Drug
**Dose Formulation**	Tablet	Tablet
**mg/tablet**	100mg/tablet	nirmatrelvir 150mg/tablet, ritonavir 100mg/tablet
**Dosage**	600mg Q12H × 1day<br>300mg Q12H × 4 days	nirmatrelvir tablet 300mg + ritonavir tablet 100mg Q12H × 5 days
**Route of Administration**	Oral administration	
**IMP and NIMP/AxMP**	IMP	IMP
**Packaging and Labeling**	Study intervention will be provided in container. Each container will be labeled as required per country requirement.	

Abbreviations: AxMP=auxiliary medicinal product, IMP = investigational medicinal product, Q12H=every 12 hours.

The study drugs will be repackaged to remove drug information, and will only include study abbreviations, drug numbers, quantities, treatment times, and methods. Investigators are not aware of the treatment assignment until after study completion and final database lock.

Unblinding: This is a single-blind study, where the treatment assignment is blinded to healthcare providers/investigators. The IWRS programming will be used for unblinding instructions. In emergency situations, only the investigators are responsible for

determining whether it is necessary to unblind the intervention assignment of a participant. When making such decisions, the safety of the participants should always be the primary consideration. If the intervention assignment of a participant is unblinded, the date and reason for unblinding must be recorded.

6.6 Study Drug Preparation/Handling/Storage/Responsibility

The study drugs will be provided by the corresponding pharmaceutical company. Expired or unused drugs will be recycled according to the specified procedures. Drug tablets are re-packaged to suit the study intervention requirement.

Please refer to the link for information of VV116:

https://drugs.dxy.cn/pc/drug/20o0dNycYCSu432DPpBHXQ==?ky=%E6%B0%91%E5%BE%97%E7%BB%B4

Please refer to the link for information of nirmatrelvir–ritonavir (Paxlovid):

https://www.accessdata.fda.gov/drugsatfda_docs/label/2023/217188s000lbl.pdf

6.7 Follow-up and Compliance

After randomization, study participants will receive the study treatment for a duration of 5 days and subsequently undergo regular nucleic acid sampling and testing for a total of 60 days. The entire study process includes screening visits, baseline visits, and 17 follow-up visits. Please refer to the ‘Schedule of assessments (SoA)’ table for details.

Communication with study participants regarding clinical symptoms and antigen test results will be conducted online during the intervention and follow-up period to enhance compliance. Compliance will be strengthened through incentives such as covering the costs of follow-up examinations and reimbursing transportation expenses for follow-up visits.

6.8 Study Intervention Commitment

The study aims to compare the viral load rebound rate in patients with mild-to-moderate COVID-19 treated with either VV116 or nirmatrelvir–ritonavir. Antiviral treatment will be strictly conducted according to the drug usage guidelines. Regular telephone follow-up will be conducted to obtain information on the patients' clinical symptoms and antigen test results to assess improvement in symptoms and potential viral clearance after drug use. Nucleic acid sampling and testing will be performed before the start of the drug treatment intervention and on the day after the intervention ends, as well as during regular follow-up visits, to determine the occurrence of viral load rebound after treatment.

6.9 Study Endpoints Assessment

6.9.1 Primary endpoint: Viral load rebound (VLR) rate within 60 days after randomization.

The definition of VLR: After completing the 5-day course of treatment with either VV116 or nirmatrelvir–ritonavir, the viral load measured by RNA copy numbers per mL during the follow-up period increased by more than 0.5 $\log_{10}$ copies/mL compared to the results obtained the day after treatment completion.

The absolute quantification of viral nucleic acid copy numbers will be measured with droplet digital PCR (instrument: dPCR-DQ24) at Shanghai Decoding Medical Laboratory.

6.9.2 Secondary endpoints

- The occurrence of a reduction in Ct value of ≥1.5 on any of the follow-up days until day 60 compared with day 6; when the RT-qPCR result was negative, Ct value was not available and was imputed with a value of 40, which was the detection limit of the assay;
- Time until VLR from completion of treatment;
- The occurrence of VLR at 7, 14, 21, 28 days after completion of treatment;
- The occurrence of symptom aggregation, defined as an increase of >2 points in total symptom score on any of the follow-up days until day 60 compared with day 6;
- The occurrence of sustained symptom aggregation, defined as an increase of >2 points in total symptom score on 2 consecutive follow-up visits until day 60 compared with day 6.

COVID-19 related symptom scores

Study personnel will ask participants questions to fill out a COVID-19 symptom score sheet (see table below) after they are enrolled before starting the study treatment. Subsequent assessments will be conducted according to the follow-up plan, at the same time on the follow-up days. Study personnel will fill out the COVID-19 symptom score sheet based on the participant's most severe symptoms within the past 24 hours. Symptom aggregation is defined as an increase of more than 2 points in the total score of COVID-19-related symptoms compared to the day after treatment completion. Sustained symptom aggregation is defined as an increase of more than 2 points in the total score of COVID-19-related symptoms compared to the day after treatment completion on 2 consecutive follow-up visits.

Table. COVID-19 related symptom score

1. Fever*	None=0<br>Mild=1<br>Moderate=2<br>Severe=3
2. Chills or shivering	
3. Headache	
4. Muscle pain	
5. Joint pain	
6. Cough	
7. Expectoration	
8. Sore throat	
9. Dry throat	
10. Sneezing	
11. Stuffy or running nose	
12. Voice hoarse	
13. Chest pain	
14. Shortness of breath or difficulty in breathing	
15. Nausea	
16. Abdominal pain	
17. Dizziness	
18. Tinnitus	
19. Earache	
20. Hypogeusia	
21. Hyposmia	
22. Vomiting	none=0, 1-2 time(s)=1, 3-4 times=2, 5 times or more=3
23. Diarrhea (unformed or watery stool)	none=0, 1-2 time(s)=1, 3-4 times=2, 5 times or more=3

* Mild fever is considered when the temperature is between 37.3℃ and 38℃, moderate fever is between 38.1℃ and 39℃, and high fever is considered when the temperature is equal to or greater than 39.1℃. The temperature measured in the mouth is approximately 0.4℃ higher than the temperature measured in the ear or 0.5℃ higher than the temperature measured in the armpit.

The criteria for determining the severity of fever may vary depending on the diagnostic instrument.

6.10 Safety and other assessments

All the planned time points for safety and other assessments could be found in the SoA. The definitions of Adverse Events (AE) and Serious Adverse Events (SAE) could be found in the Appendix. Investigators and any designated personnel are responsible for detecting, archiving, and documenting events that meet the definitions of AE or SAE, and for following up on all AEs. The recording, evaluation, and assessment methods for the causal relationship of AE and SAE, as well as the procedures for filling out and sending SAE reports, could be found in the Appendix.

All AEs and SAEs will be collected from the signing of the informed consent form (ICF) until the follow-up visit at the timepoints specified in the SoA. All SAEs will be recorded and reported to the IRB immediately and under no circumstance should this exceed 24 hours, as indicated in the Appendix. The investigator will submit any updated SAE data to the IRB within 24 hours of it being available.

Investigators are not obligated to actively seek information on AEs or SAEs after conclusion of the study. However, if the investigator learns of any SAE, including a death, at any time after a participant has been discharged from the study, and he/she considers the event to be reasonably related to the study intervention or study participation, the investigator must promptly notify the IRB.

6.11 Sample Size Determination

The sample size calculation is based on the primary outcome of the study, which is the viral load rebound rate in COVID-19 infected patients after study drug treatment and within 60 days of randomization. The significance level for the two-sided test (α) is set at 5%, with a statistical power of 80%. The table below shows the anticipated sample size for a single group under different scenarios of viral load rebound rate in the nirmatrelvir–ritonavir treatment group and the difference in viral load rebound rate between the treatment groups.

Table. Sample size calculation

	The difference of VLR rate between the treatment groups						
VLR rate in nirmatrelvir–ritonavir treatment group	-0.02	-0.03	-0.04	-0.05	-0.06	-0.07	-0.08
0.05	1605	653	333				
0.08	2653	1125	602	365	**239**	164	
0.10	3313	1422	771	474	316	222	162
0.12	3941	1704	932	578	389	276	204
0.15	4824	2102	1158	726	493	353	264
0.17	5373	2350	1300	817	557	401	301
0.20	6139	2695	1497	945	647	469	354
0.23	6833	3009	1677	1062	730	530	401
0.25	7257	3201	1787	1134	781	568	431

We assume that the VLR rate in the nirmatrelvir–ritonavir group is 8% and there will be a 6% reduction in VLR rate in the VV116 treatment group. A total of 478 participants needs to be enrolled with 239 participants in the VV116 treatment group and 239 in the nirmatrelvir–ritonavir treatment group.

Table. Schedule of assessments (SoA)

**Procedure**	**Scr** [1]	**Bs/Rz**	**Treatment and assessment** [2]																		**E/D**
**Visits**	**V1**	**V2**	**5-day treatment**	**V3**	**V4**	**V5**	**V6**	**V7**	**V8**	**V9***	**V10**	**V11***	**V12**	**V13***	**V14**	**V15**	**V16**	**V17**	**V18***	**V19**	**V20**	**V21**
**Study day**	**-5 ~ -1**	**0**	**1 ~ 5**	**6**	**8**	**10**	**12**	**14**	**16**	**18***	**20**	**22***	**24**	**26***	**28**	**35**	**42**	**49**	**56***	**60**	**VLR day**	
**Visit window (days)**	**-**	**-**	**-**	**+1**	**±1**	**±1**	**±1**	**±1**	**±1**	**±1**	**±1**	**±1**	**±1**	**±1**	**±1**	**±3**	**±3**	**±3**	**+3**	**+3**	**+1**	**±1**
Informed consent	X																					
Inclusion and exclusion criteria [3]	X																					
Demographics [4]	X																					
Height and weight[5]	X																					
Pre-existing conditions and current medical history [6]	X																					

Medical history for COVID-19 [7]	X																					
Prior SARS-CoV-2 vaccination or preventive antibody treatment [8]	X																					
Prior treatment of COVID-19 [9]	X																					
Laboratory tests and blood sample collection [10]		X		X																X	X	X
Oropharyngeal swab sampling and SARS-CoV-2 nucleic acid testing [11]		X		X	X	X	X	X	X	X	X	X	X	X	X	X	X	X	X	X	X	X
Assessment of symptoms [12]		X		X	X	X	X	X	X	X	X	X	X	X	X	X	X	X	X	X	X	X
Adverse events			X	X	X	X	X	X	X	X	X	X	X	X	X	X	X	X	X	X	X	X

Concomitant medications		X	X	X	X	X	X	X	X	X	X	X	X	X	X	X	X	X	X	X	X	X

* The collection of oropharyngeal swabs will be collected by study nurses blinded to treatment assignment. If the designated nucleic acid sampling cannot be completed on these visit days, it will not be considered a violation of the protocol.

1. Screening, baseline examination, and randomization can be conducted on the same day.
2. Non-institutionalized /discharged participants can be interviewed by phone, and the content of the interview can be adjusted according to the patient's condition.
3. Recheck the clinical condition before randomization to confirm the inclusion/exclusion criteria.
4. Including age and gender.
5. To calculate body-mass index (BMI).
6. Obtained from inquiry or available information.
7. Including COVID-19 diagnosis, nucleic acid positive date, COVID-19 symptom onset date, and previous symptoms.
8. SARS-CoV-2 vaccination or receive of prophylactic antibodies at any times.
9. Within the last 30 days: COVID-19 antiviral drugs, other antiviral drugs, NSAIDs, antibiotics, glucocorticoids, or other drugs.
10. During screening, test results of blood routine and blood biochemistry tests conducted within the last 30 days are acceptable.
    - Blood routine: white blood cell count, lymphocyte count, lymphocyte percentage, neutrophil count, neutrophil percentage, monocyte count, monocyte percentage, red blood cell count, hemoglobin, hematocrit, platelet count;
    - Blood biochemistry: alanine aminotransferase, gamma-glutamyl transferase, aspartate aminotransferase, alkaline phosphatase, total bilirubin, direct bilirubin, total protein, albumin, urea/urea nitrogen, creatinine, uric acid.
11. If study medication starts on the day of randomization, i.e., one dose on D0, treatment will be completed in the morning of the 5th day, and blood and nucleic acid sampling can be arranged in the afternoon of the same day or in the morning of the next day.
12. The investigator will contact the participant and complete the COVID-19-related symptom scoring form at approximately the same time on each visit day.

Abbreviations: Bs/Rz = baseline and randomization visits; COVID-19 = Coronavirus Disease 2019; E/D = Early Discontinuation; NSAIDs = Non-Steroidal Anti-Inflammatory Drugs; SARS-CoV-2 = Severe Acute Respiratory Syndrome Coronavirus 2; Scr = Screening; VLR = viral load rebound

7. Discontinuation of Study Intervention and Participant Withdrawal

7.1 Discontinuation of Study Intervention

In rare instances, it may be necessary for a participant to permanently discontinue study intervention (e.g., If at the discretion of the investigator, continuation of study intervention is exposing the participant to an unacceptable level of risk), study intervention is permanently discontinued, and the participant will remain in the study to be evaluated for the remainder of the assessment visits. See the SoA for data to be collected at the time of discontinuation of study intervention and follow-up and for any further evaluations that need to be completed.

Discontinuation of study intervention for abnormal liver tests is required by the investigator when they meet any of the following stopping rules, or in the presence of abnormal liver test results, judged by the investigator that it is in the best interest of the participant, even though they might not meet protocol-specified stopping rules. A repeated liver test should be taken in 24-48 hours to confirm the test result.

- ALT or AST ≥ 8 × ULN (or baseline, if abnormal at baseline)
- ALT or AST ≥ 3 × ULN (or baseline if abnormal at baseline) and total bilirubin (TBL) ≥ 2 × ULN (or baseline if abnormal at baseline)

7.2 Participant Discontinuation/Withdrawal from the Study

- A participant may withdraw from the study at any time at his/her own request or may be withdrawn at any time at the discretion of the investigator for safety, behavioral, or compliance reasons. This is expected to be uncommon.
- At the time of discontinuing from the study, if possible, an early discontinuation visit should be conducted, as shown in the SoA. See SoA for data to be collected at the time of study discontinuation and follow-up and for any further evaluations that need to be completed.
- The participant will be permanently discontinued from the study intervention and the study at that time.
- If the participant withdraws consent for disclosure of future information, the research team may retain and continue to use any data collected before such a withdrawal of consent.
- If a participant withdraws from the study, he/she may request destruction of any samples taken and not tested, and the investigator must document this in the study records.

7.3 Loss to Follow-up

A participant will be considered lost to follow-up if she/he repeatedly fails to return for scheduled visits and is unable to be contacted by the study personnel.

The following actions must be taken if a participant fails to return to the clinic for a required study visit:

- The site must attempt to contact the participant and reschedule the missed visit as soon as possible, counsel the participant on the importance of maintaining the assigned visit schedule, and ascertain whether the participant wishes to and/or should continue in the study.
- Before a participant is deemed lost to follow-up, the investigator must make every effort to regain contact with the participant (where possible, 3 telephone calls, and if necessary, a certified letter to the participant's last known mailing address or local equivalent methods). These contact attempts should be documented in the participant's medical record.
- Should the participant continue to be unreachable, he/she will be considered to have withdrawn from the study.

Study personnel will attempt to collect the vital status of the participant within legal and ethical boundaries for all participants randomized, including those who did not get study intervention. Public sources may be searched for vital status information. If vital status is determined as deceased, this will be documented and the participant will not be considered lost to follow-up.

8. Statistical Analyses

8.1 Analysis Set

For the purposes of analysis, the following analysis sets are defined:

(1) Full analysis set (FAS): All randomized patients who received at least one dose of the study drug and had at least one follow-up visit in addition to D6.
(2) Per-protocol Set (PPS): A subset of FAS. All participants who took at least 80% of the required doses of the study drug and without any major protocol violations such as taking other anti-SARS-CoV-2 drugs during study intervention.
(3) The Safety Set (SS): All participants who received at least one dose of the study drug and had at least one post-dose safety assessment.

All the primary outcome and secondary outcome data will be analyzed using the FAS. PPS will be used to analyze the endpoint related to the primary objective as a sensitivity analysis. SS will be used to analyze safety-related endpoints.

8.2 Primary Endpoint Analysis

Statistical assumptions

H0: π1 = π2, the VLR rates of VV116 and nirmatrelvir–ritonavir after completing treatments within 60 days for COVID-19 patients are the same.

H1: π1 ≠ π2, the VLR rates of VV116 and nirmatrelvir–ritonavir after completing treatments within 60 days for COVID-19 patients are different.

The primary endpoint analysis will be conducted based on the FAS set. The comparison of VLR rates within 60 days after randomization between the two groups will be performed using the Chi-square test (or Fisher's exact test if the conditions for Chi-square test are not met. The Fisher's exact test will be used when the total number of primary endpoint is less than 40, when the theoretical frequency of any category in the fourfold table is less than 5, or when the P-value from the Chi-square test is approximately equal to the significance level [α]).

8.3 Secondary Endpoints Analysis

- Time until VLR from completion of treatment (days)

The Kaplan-Meier method will be used to estimate the median time of VLR for each group, and the 95% CI will be estimated using the BrookMeyer-Crowley method with log-log transformation.

- The percentage of cases in which the semi-quantitative level of viral nucleic acid Ct value decreased by more than 1.5 compared with the day after treatment completion during the follow-up.
- The percentages of participants experiencing VLR within 7, 14, 21, 28 days after completion of treatment.
- The percentage of participants who experienced symptom aggregation, defined as an increase of >2 points in total symptom score on any of the follow-up days compared with that on the day after treatment completion.
- The percentage of participants who experienced sustained symptom aggregation, defined as an increase of >2 points in total symptom score on 2 consecutive follow-up visits compared with that on the day after treatment completion.

The analytical method for the above endpoints is the same as that of the analysis for the primary endpoint.

8.4 Safety Analysis

The safety assessment up to day 60 will be analyzed, including AE and SAE. Analyses of safety will be summarized based on SS. Terms including AEs and medical history will be coded using the Medical Dictionary for Regulatory Activities (MedDRA) version 24.1 or higher. TEAEs (Treatment Emergent Adverse Event, TEAE) will be

listed, and if the frequency of events allows, will be summarized using descriptive methodology. The incidence of TEAEs for each treatment will be presented by severity and by association with intervention as perceived by the investigator.

Safety parameters that will be assessed including laboratory parameters, and vital signs, etc. The parameters will be listed and summarized using descriptive statistics. Additional analysis will be performed if warranted upon review of the data.

8.5 Baseline Descriptive Analyses

For continuous variables, descriptive statistics will include mean ± standard deviation for normal-distributed data and quartiles (including the median, first quartile, and third quartile for skewed-distributed data). For categorical variables, descriptive statistics will include the number of subjects, number of events, and percentages. Unless otherwise specified, the denominator for calculating percentages will be the total number of participants by treatment groups.

8.6 Subgroup Analysis

The subgroup analysis will be performed on the primary endpoint to evaluate the consistency in intervention effects between the following subgroups:

- Age group: < 40 years; 40-60 years; ≥ 60 years
- Gender: women; men
- BMI: ≥ 25 kg/m^2; < 25 kg/m^2
- Diabetes: with; without
- Cardiovascular diseases (including hypertension): with; without
- SARS-CoV-2 vaccination: unvaccinated; vaccinated (no booster); vaccinated (booster)
- Time from first positive test for SARS-CoV-2 to first dose of study drugs: < 3 days; ≥ 3 days

9. Supporting Documentation and Operational Considerations

9.1 Informed Consent Process

Investigators must ensure that participants are provided with complete and adequate verbal and written information about the nature, purpose, potential risks, and benefits of the study. Participants must also be informed that they have the right to withdraw from the study at any time. They should be given the opportunity to ask questions

and given time to consider the information provided. Before proceeding with any specific procedures of the study, informed consent from participants must be obtained in writing and signed and dated, including: interviews with investigators, filling out questionnaires, completing CRFs, etc. Investigators must keep the original copies of signed informed consent forms from participants. Investigators must provide participants with a signed copy of the informed consent form.

9.2 Privacy Policy

Without the consent of the principal investigator, investigators are prohibited from disclosing any information of this experimental protocol to third parties, and this confidentiality obligation remains in effect even after the trial is terminated or completed. Investigators and their staff collect personal data of the participants for this research (‘research data’), including their date of birth, gender, contact information, and personal data related to their disease and health status. All identifiable participants' cases and research data will be kept confidential within the limits allowed by law. However, investigators have the right to disclose identifiable confidential information in certain specific circumstances and when inspected and copied by the ethics committee. All personal information collected during the study will be processed in accordance with national and local data protection laws.

Participants have the right to request investigators to protect their relevant information and have the right to correct any inaccuracies in the data. If a participant withdraws their informed consent, investigators will no longer use the participant's data or disclose them to others, but they may continue to use the research data obtained prior to the withdrawal of consent.

The results of this study may be published in medical journals and presented at medical conferences. In any publication or conference, the identity of the participants will not be disclosed without their consent.

9.3 Collection and Use of Biological Samples and Data

Biological specimens at Ruijin Hospital are tested in accordance with biosafety regulations in the laboratory. All clinical data are retained and stored by Ruijin Hospital, in both paper and electronic formats.

After the completion of the study, all data and other information can be used for future research with the consent of the research participants and the principal investigator. The data are stored in encrypted computers.

Collection and storage of oropharyngeal swab specimens: oropharyngeal swab specimens are collected using non-inactivated sampling tubes, sealed in double-layer bags, and stored at 4°C. After sample collection, samples are taken by designated

personnel every day at specified timepoints and frequencies as stated in the SoA. Samples will be stored in a -80°C freezer for future research after assay.

Collection and storage of blood specimens: biological specimens are collected at specified timepoints and frequencies as stated in the SoA. The collected specimens are placed on ice or stored at 4°C before clinical testing.

9.4 Data Quality Control and Assurance

The clinical research center is responsible for the quality control and assurance of the study data.

Before recruiting the first participant, make sure the relevant facilities and research materials are sufficient, and that the investigators have the ability to recruit the eligible participants. Discuss with the investigators (and other personnel involved in the study) their responsibilities in terms of compliance with the protocol. During the study, provide information and support to the investigators. Confirm whether the research team is following the study protocol and ensure that the relevant data are accurately recorded in the case report forms (CRFs). Ensure that the participants have signed informed consent forms, which are kept at the research center. Ensure that the CRFs are completed appropriately, timely, and with high quality. Check the medical records and source documents of the participants for verification. The principal investigator must ensure that the relevant research personnel have received appropriate training related to the study.

9.5 Data Processing and Record Keeping

9.5.1 Data Collection and Management

According to the 'Quality Management Specification for Clinical Trials of Medical Devices' source data and source files are defined as follows:

A. Source data refer to the original records of clinical observations, findings, and other activities in a clinical trial, as well as all information in their approved copies, which can be used for the reconstruction and evaluation of the clinical trial.

B. Source files refer to printed documents, visual files, electronic files, or other forms that contain source data.

The data in this study are primarily collected through CRF forms, which must be filled out promptly and accurately. Any missing or abnormal data must be explained. The laboratory test results involved in this study will be accompanied by specific test reports and recorded in the CRF forms. In addition, a cloud database will be established specifically for storing the raw data from the CRF forms. All research

personnel will have only one account with password protection. The account password should not be shared with any unrelated individuals without the consent of the principal investigator of the study.

All completed CRF forms must be reviewed and signed by the investigators. The data manager has access to the data in the CRF forms for data management purposes. The statisticians can access the data in the CRF forms for statistical analysis. The monitors can verify the accuracy, timeliness, and completeness of the data in the CRF forms based on the source data, and ensure that all missing or abnormal data are resolved.

9.5.2 Data retention

After completing the study, investigators must comply with local laws and regulations to keep study files, patient medical records, and case reports for at least 2 years (or longer). Investigators should use various measures such as safe boxes, sealing, and electronic backups to prevent accidental or premature destruction of clinical research materials. Permission from the principal investigator should be obtained before disposing of clinical research materials.

9.6 Publication and Data Sharing Agreement

This study is registered on a public website and results of the study will be published in a scientific journal.

9.7 Statement of Conflict of Interest

None.

Appendix：AEs and SAEs: Definitions and Procedures for Recording, Evaluating, Follow-up, and Reporting

Definition of AE

AE Definition
• According to the Good Clinical Practice (GCP) guidelines for the quality management of drug clinical trials, an AE is any untoward medical occurrence in a clinical study participant, temporally associated with the use of study intervention, whether or not considered related to the study intervention.<br>• NOTE: An AE can therefore be any unfavorable and unintended sign (including an abnormal laboratory finding), symptom, or disease (new or exacerbated) temporally associated with the use of study intervention.
**Events Meeting the AE Definition**
• Any abnormal laboratory test results (hematology, clinical chemistry, or urinalysis) or other safety assessments (e.g., ECG, radiological scans, vital signs measurements), including those that worsen from baseline, considered clinically significant in the medical and scientific judgment of the investigator (i.e., not related to progression of underlying disease)<br>• Exacerbation of a chronic or intermittent pre-existing condition including either an increase in frequency and/or intensity of the condition<br>• New condition detected or diagnosed after study intervention administration even though it may have been present before the start of the study<br>• Signs, symptoms, or the clinical sequelae of a suspected intervention-intervention interaction<br>• Signs, symptoms, or the clinical sequelae of a suspected overdose of either study intervention or a concomitant medication (overdose itself is not reported as an AE/SAE)<br>• Lack of efficacy or failure of expected pharmacological action per se will not be reported as an AE or SAE. Such instances will be captured in the efficacy assessments. However, the signs, symptoms, and/or clinical sequelae resulting from lack of efficacy will be reported as AE or SAE if they fulfill the definition of an AE or SAE.
**Events NOT Meeting the AE Definition**

• The following study-specific clinical events related to COVID-19 are exempt from adverse event reporting unless the investigator deems the event to be related to the administration of study drug:<br>o Hypoxemia due to COVID-19 requiring supplemental oxygen；<br>o Hypoxemia due to COVID-19 requiring non-invasive ventilation or high flow oxygen devices；<br>o Respiratory failure due to COVID-19 requiring invasive mechanical ventilation or ECMO<br>• Any clinically significant abnormal laboratory findings or other abnormal safety assessments that are associated with the underlying disease, unless judged by the investigator to be more severe than expected for the participant's condition<br>• The disease/disorder being studied or expected progression, signs, or symptoms of the disease/disorder being studied, unless more severe than expected for the participant's condition<br>• Medical or surgical procedure (e.g., endoscopy, appendectomy): the condition that leads to the procedure is the AE, like appendicitis<br>• Situations in which an untoward medical occurrence did not occur (social and/or convenience admission to a hospital)<br>• Anticipated day-to-day fluctuations of pre-existing disease(s) or condition(s) present or detected at the start of the study that do not worsen

Definition of SAE

**An SAE is defined as any untoward medical occurrence that meets one or more of the criteria listed:**
**a. Results in death**
**b. Is life threatening**<br>The term life threatening in the definition of SAE refers to an event in which the participant was at risk of death at the time of the event. It does not refer to an event, which hypothetically might have caused death, if it were more severe.
**c. Requires inpatient hospitalization or prolongation of existing hospitalization**<br>• In general, hospitalization signifies that the participant has been admitted (usually involving at least an overnight stay) at the hospital or emergency ward for observation and/or treatment that would not have been appropriate in the

**An SAE is defined as any untoward medical occurrence that meets one or more of the criteria listed:**
physician's office or outpatient setting. Complications that occur during hospitalization are AEs. If a complication prolongs hospitalization or fulfills any other serious criteria, the event is serious. When in doubt as to whether hospitalization occurred or was necessary, the AE should be considered serious.<br>• The following specific hospitalizations are not required to be reported as SAE:<br>o Hospitalization for elective treatment of a pre-existing disease that has not worsened from baseline;<br>o Planned hospitalization as required in the protocol;<br>o Pre-study planned hospitalization or elective surgery not due to adverse events;<br>o Hospitalization only for temporary nursing reasons, but no adverse events occurred;<br>o Hospitalization due to other living conditions, not related to health status (such as medical insurance reimbursement, and management reasons);<br>o Hospitalization only for blood product use, not caused by adverse events.
**d. Results in persistent or significant disability/incapacity**<br>• The term disability means a substantial disruption of a person's ability to conduct normal life functions.<br>• This definition is not intended to include experiences of relatively minor medical significance such as uncomplicated headache, nausea, vomiting, diarrhea, influenza, and accidental trauma (e.g., sprained ankle) that may interfere with or prevent everyday life functions but do not constitute a substantial disruption.
**e. Is a congenital anomaly/birth defect**
**f. Other important medical events, if do not receive any treatments, this is a possibility of experiencing the aforementioned conditions:**<br>Medical or scientific judgment should be exercised by the investigator in deciding whether SAE reporting is appropriate in other situations such as significant medical events that may jeopardize the participant or may require medical or surgical intervention to prevent one of the other outcomes listed in the above definition. These events should usually be considered serious.

An SAE is defined as any untoward medical occurrence that meets one or more of the criteria listed:
o Examples of such events include invasive or malignant cancers, intensive treatment for allergic bronchospasm, blood dyscrasias, convulsions or development of intervention dependency or intervention abuse.

Recording and Follow-Up of AE and/or SAE

AE and SAE Recording
• When an AE/SAE occurs, it is the responsibility of the investigator to review all documentation (e.g., hospital progress notes, laboratory reports, and diagnostic reports) related to the event.<br>• The investigator will then record all relevant AE/SAE information.<br>• It is not acceptable for the investigator to send photocopies of the participant's medical records to Sponsor in lieu of completion of the AE/SAE CRF page/required form.<br>• There may be instances when copies of medical records for certain cases are requested by Sponsor. In this case, all participant identifiers, with the exception of the participant number, will be redacted on the copies of the medical records before submission to Sponsor.<br>• The investigator will attempt to establish a diagnosis of the event based on signs, symptoms, and/or other clinical information. Whenever possible, the diagnosis (not the individual signs/symptoms) will be documented as the AE/SAE.

Assessment of Causality
• The investigator is obligated to assess the causal relationship between study intervention and each occurrence of each AE/SAE. The investigator will use clinical judgment to determine the relationship.<br>• A reasonable possibility of a relationship conveys that there are facts, evidence, and/or arguments to suggest a causal relationship, rather than a relationship cannot be ruled out.<br>• Alternative causes, such as underlying disease(s), concomitant therapy, and other risk factors, as well as the temporal relationship of the event to study intervention administration, will be considered and investigated.

**Assessment of Causality**
• The investigator will also consult the investigator's brochure (IB) and/or product information, for marketed products, in his/her assessment.<br>• For each AE/SAE, the investigator **must** document in the medical notes that he/she has reviewed the AE/SAE and has provided an assessment of causality.<br>• There may be situations in which an SAE has occurred and the investigator has minimal information to include in the initial report to Sponsor. However, it is very important that the investigator always make an assessment of causality for every event before the initial transmission of the SAE data to Sponsor.<br>• The investigator may change his/her opinion of causality in light of follow-up information and send an SAE follow-up report with the updated causality assessment.<br>• The causality assessment is one of the criteria used when determining regulatory reporting requirements.<br>• The investigator will assess and document the causal relationship between investigational study drug and each AE by answering the question "Do you consider that there is a reasonable possibility that the event may have been caused by the study treatment?"<br>• An answer of "yes" indicates that there is a reasonable suspicion that the AE is associated with the use of the study treatment.<br>• Factors to be considered in assessing the relationship of the AE to study treatment include:<br>o The temporal sequence from drug administration: The event should occur after the drug is given. The length of time from drug exposure to event should be evaluated in the clinical context of the event.<br>o Recovery on drug discontinuation (de-challenge), recurrence on drug re-introduction (re-challenge):<br>Patient's response after de-challenge or patient's response after re-challenge should be considered in the view of the usual clinical course of the event in question.<br>o Underlying, concomitant, intercurrent diseases:<br>Each event should be evaluated in the context of the natural history and course of the disease being treated and any other disease the patient may have.

Assessment of Causality
o Concomitant medication or treatment:<br>The other drugs the patient is taking or the treatment the patient receives should be examined to determine whether any of them may be suspected to cause the event in question.<br>• The pharmacology and pharmacokinetics of the study treatment: The pharmacokinetic properties (absorption, distribution, metabolism and excretion) of the study treatment, coupled with the individual patient's pharmacodynamics should be considered.<br>• An assessment of "no" indicates that there is non-plausibility or the existence of a clear alternative explanation.

Follow-up of AEs and SAEs
• The investigator is obligated to perform or arrange for the conduct of supplemental measurements and/or evaluations as medically indicated or as requested by Sponsor to elucidate the nature and/or causality of the AE or SAE as fully as possible. This may include additional laboratory tests or investigations, histopathological examinations, or consultation with other health care professionals.<br>• If a participant dies during participation in the study or during a recognized follow-up period, the investigator will provide Sponsor with a copy of any postmortem findings including histopathology, if applicable.<br>• The investigator will submit any updated SAE data to Sponsor within 24 hours of receipt of the information.

Reporting of SAEs/Pregnancy

SAE Reporting to Sponsor via SAE/Pregnancy Report Form
• Investigators must fill the SAE/Pregnancy report form provided by Sponsor and report to the Sponsor within 24 hours of becoming aware of the SAE/Pregnancy, regardless of relationship to the study medication.<br>• Contacts for SAE/Pregnancy reporting can be found in site training documents.

Ruijin Hospital, Shanghai Jiaotong University School of Medicine

Statistical Analysis Plan

Study Title：	COVID-19 rebound after JT001 (VV116) and nirmatrelvir–ritonavir treatment in patients with mild to moderate COVID-19: A single-blind, parallel-group, randomized clinical trial
Principal Investigator：	Guang Ning, Yufang Bi, Yiping Xu
Study Start：	December 2022 ~

Version Number: 1.0

January 5th, 2023

TABLE OF CONTENTS

TABLE OF CONTENTS **2**
LIST OF ABBREVIATIONS **4**
1 INTRODUCTION **5**
1.1 Objectives and Endpoints 5
1.2 Study Design 6
2 STATISTICAL HYPOTHESIS **6**
3 SAMPLE SIZE ESTIMATION **6**
4 RANDOMIZATION, BLINDING, AND PARTICIPANT REPLACEMENT **7**
4.1 Randomization 7
4.2 Blinding and Unblinding 7
4.3 Participant Replacement 8
5 STATISTICAL ANALYSIS **8**
5.1 General Principles 8
5.1.1 Definition of Baseline 8
5.2 Statistical Analysis Sets 8
5.3 Analysis Population 9
5.3.1 Disposition information 9
5.3.2 Protocol deviations 9
5.4 Demographics and Baseline Characteristics 9
5.4.1 Demographic characteristics 9
5.4.2 Medical history and symptom scoring 9
5.5 Prior/Concomitant Drug Therapy and Non-Drug Therapy 9
5.5.1 History of SARS-CoV-2 vaccine or prophylactic antibodies 10
5.5.2 Prior drug and non-drug therapies for COVID-19 10
5.6 Efficacy Analysis 10
5.6.1 Primary efficacy endpoint 11
5.6.2 Secondary efficacy endpoints 11
5.7 Safety Analysis 12
5.7.1 Drug exposure and compliance 12
5.7.2 Adverse event 12
5.7.3 Death 12
5.7.4 Laboratory tests 12
5.8 Subgroup Analysis 12

5.9 Interim Analysis 13
6 APPENDIX 1 14

LIST OF ABBREVIATIONS

Abbreviation/Term	Definition/Description
AE	Adverse event
COVID-19	Coronavirus disease 2019
CRF	Case report form
FAS	Full analysis set
ICF	Informed consent form
IDMC	Independent data monitoring committee
LOCF	Last observation carried forward
MedDRA	Medical Dictionary for Regulatory Activities
PPS	Per protocol set
PT	Preferred term
SAE	Serious adverse event
SARS-CoV-2	Severe acute respiratory syndrome coronavirus 2
SAP	Statistical analysis plan
SOC	System organ class
SS	Safety set
TEAE	Treatment-emergent adverse event
VLR	Viral load rebound
WHO	World Health Organization

1 INTRODUCTION

This Statistical Analysis Plan (SAP) describes the statistical analysis methods and the form of result presentation regarding 'COVID-19 rebound after JT001 (VV116) and nirmatrelvir–ritonavir treatment in patients with mild to moderate COVID-19: A single-blind, parallel-group, randomized clinical trial (version 1.0; date: Jan. 5th, 2023)'.

The scope of this SAP includes comprehensive and detailed statistical strategies, principles, and methods for assessing the post-treatment viral load rebound (VLR) rate in mild-to-moderate COVID-19 patients after receiving treatments.

1.1 Objectives and Endpoints

Objectives	Endpoints
**Primary objectives**	**Primary endpoints**
Compare the incidence of VLR among mild-to-moderate COVID-19 patients treated with VV116 or nirmatrelvir–ritonavir	The VLR rate within 60 days after randomization (VLR defined as an increase in quantitative RT-PCR viral load by >0.5 log_{10} copies/mL compared with the day after treatment completion)
**Secondary objectives**	**Secondary endpoints**
Compare the incidence rate of semi-quantitative level of viral nucleic acid Ct value decrease in mild-to-moderate COVID-19 patients treated with VV116 or nirmatrelvir–ritonavir	The percentage of cases in which the viral nucleic acid Ct value decreased by more than 1.5 compared with the day after treatment completion
Assess the effect on the viral rebound time of VV116 compared to nirmatrelvir–ritonavir	Time until VLR from treatment completion (days)
Evaluate the occurrences of VLR at different time intervals after treatment completion	The occurrences of VLR at 7, 14, 21, and 28 days after treatment completion
Evaluate the recurrences of COVID-19 related symptoms after treatment completion	Percentages of participants with symptom aggregation and sustained symptom aggregation during the follow-up period until 60 days after randomization compared with that on the day after treatment completion
**Safety**	**The safety assessment results (e.g., SAE) as of Day 60**

*Note: Definition of symptom aggregation: an increase of >2 points in total symptom score on any of the follow-up days until 60 days compared with that on the day after treatment completion.

Definition of sustained symptom aggregation: an increase of >2 points in total symptom score on 2 consecutive follow-up visits until 60 days compared with that on the day after treatment completion.

1.2 Study Design

This is a single-center, single-blind, randomized, controlled trial to evaluate the occurrence of VLR of JT001 (VV116) compared with nirmatrelvir–ritonavir for the treatment of mild-to-moderate COVID-19.

The participants will undergo all study procedures only after signing the applicable informed consent form (ICF).

Before any invasive procedure, investigators will review the clinical symptoms, risk factors, and other non-invasive inclusion and exclusion criteria. Participants passing the review will undergo invasive procedures performed by the study site to confirm their eligibilities.

Intervention groups and duration:

**Group**	**Intervention**	**Dose regimen**	**Administration route**
Group 1	JT001 (VV116)	JT001 (VV116) 600 mg Q12H × 1 day<br>300 mg Q12H × 4 days	Oral
Group 2	nirmatrelvir–ritonavir (Paxlovid)	Nirmatrelvir tablets 300 mg + ritonavir tablets 100 mg, Q12H × 5 days	

Q12H = once every 12 hours

2 Statistical Hypothesis

The hypothesis is to demonstrate the difference in VLR rate among COVID-19 patients after receiving VV116 and nirmatrelvir–ritonavir treatment, respectively, within 60 days.

$$H_0: \pi1 = \pi2,\ H_a: \pi1 \neq \pi2$$

3 Sample Size Estimation

Assumptions for VLR rate：

The sample size calculation is based on the primary outcome of the study, which is the occurrence of VLR within 60 days after randomization and start receiving treatment among COVID-19 infected patients. A two-sided significance level (α) of 5% and a statistical power of 80% are set. The table below lists the sample size for a single group under different rates of VLR in the nirmatrelvir–ritonavir treatment group and the difference in VLR rate lower than the nirmatrelvir–ritonavir group in the VV116 treatment group.

	The difference of VLR rate between the treatment groups						
VLR rate in nirmatrelvir–ritonavir treatment group	-0.02	-0.03	-0.04	-0.05	-0.06	-0.07	-0.08
0.05	1605	653	333				
0.08	2653	1125	602	365	239	164	
0.10	3313	1422	771	474	316	222	162
0.12	3941	1704	932	578	389	276	204
0.15	4824	2102	1158	726	493	353	264
0.17	5373	2350	1300	817	557	401	301
0.20	6139	2695	1497	945	647	469	354
0.23	6833	3009	1677	1062	730	530	401
0.25	7257	3201	1787	1134	781	568	431

It is assumed that the VLR rate in the nirmatrelvir–ritonavir group is 8% and there will be a 6% reduction in VLR rate in the VV116 treatment group. A total of 478 participants needs to be randomly assigned. This includes 239 study participants in the VV116 treatment group and 239 participants in the nirmatrelvir–ritonavir treatment group.

4 Randomization, Blinding, and Participant Replacement

4.1 Randomization

This is a randomized, single-blind study. All participants will be assigned to the two groups in a 1:1 ratio using block randomization. After the screening process at the study center, eligible participants who meet the inclusion and exclusion criteria will be reviewed and confirmed by the coordinating center. The central randomization system will then perform the randomization process and provide the randomized group numbers for the participants.

4.2 Blinding and Unblinding

The intervention measures in this study involve the use of tablet formulations that have been repackaged and blinded. The investigators/physicians are blinded to the intervention, while blinding is not implemented for the participants. The study involves repeated nucleic acid testing on all the participants. The sampling, testing, and evaluation are conducted by investigators who are blinded to the study treatment assignment.

The investigators will not know the specific treatment assignment until the final database lock at the end of the study.

In case of emergencies, participants' safety must be with priority. Only investigators are responsible for determining the necessity to unblind the intervention assignment of participants. If a participant assignment is unblinded, the date of unblinding and the corresponding justification must be documented.

4.3 Participant Replacement

Participant replacement is not allowed in this study.

5 Statistical Analysis

5.1 General Principles

The statistical analyses will be performed using SAS 9.4 or a higher version of the statistical software.

Continuous variables will be described by descriptive statistics, including the mean, standard deviation, quartiles (including median and the 1st and 3rd quartiles). Categorical variables will be described by descriptive statistics, including the number of participants, number of events, and percentage. If a number is 0, the corresponding percentage will not be displayed. Unless otherwise specified, the denominator for calculating the percentage will be the total number of participants from the corresponding group in the analysis set.

Unless otherwise specified, the safety set (SS) will be used for the safety list and the full analysis set (FAS) for other lists, except screening failure and disposition lists.

5.1.1 Definition of Baseline

Unless otherwise specified, the baseline characteristics are defined as the results of the last assessment before taking the first dose of study medication. Please refer to Appendix 1 for the method of handling missing data.

5.2 Statistical Analysis Sets

Participant analysis set	Description
Full analysis set (FAS)	All randomized participants who received intervention and participated in follow-up visits. Participants will be analyzed according to the randomization groups.
Per protocol set (PPS)	A subset of FAS. All participants without protocol deviations that could potentially impact the primary efficacy analysis, including actual cumulative drug dose/expected cumulative drug dose ≥ 80% and no receipt of other antiviral treatments.
Safety set (SS)	All participants who received at least one dose of study medication and had at least one post-dose safety assessment. Participants will be analyzed according to the intervention they were actually received.

All the primary outcome and secondary outcome data will be analyzed using the FAS. PPS will be used to analyze the endpoint related to the primary objective as a sensitivity analysis. SS will be used to analyze safety-related endpoints.

5.3 Analysis Population

5.3.1 Disposition information

Disposition will be summarized for all randomized subjects using the categories listed below:

- Screen failure;
- Participants who are randomized;
- Participants who received intervention;
- Participants who completed the study;
- Participants who discontinued study medication;
- Participants without vital status.

The reasons for non-randomization, withdrawal from study (eg, lost to follow-up, consent withdrawal), and discontinuation from study medication will be summarized.

The percentage will be calculated based on FAS.

The corresponding listing will be provided.

5.3.2 Protocol deviations

The number of participants with major protocol deviations and their percentages will be summarized based on FAS. Additionally, all participants with protocol deviations will be presented in data listing.

5.4 Demographics and Baseline Characteristics

Unless otherwise specified, demographics and baseline characteristics will be summarized based on FAS.

Demographics and baseline characteristics will be presented in data listing.

5.4.1 Demographic characteristics

Demographic data will include participants' age (years), age group (< 60 years vs. ≥ 60 years), gender, and body mass index (kg/m^2).

5.4.2 Medical history and symptom scoring

The proportions of participants with history of disease and the mean scores of COVID-19 symptoms at randomization will be included in the table.

5.5 Prior/Concomitant Drug Therapy and Non-Drug Therapy

Unless otherwise specified, drug therapies will be coded by Anatomical Therapeutic Chemical (ATC) Classification System 2nd level and preferred name (PN) in the WHO Drug Dictionary (01Sep2021 or later), non-drug therapies will be coded by SOC and PT in MedDRA version 25.0 or higher, and the therapies will be tabulated and grouped as per the received treatment regimen.

5.5.1 History of SARS-CoV-2 vaccine or prophylactic antibodies

SARS-CoV-2 vaccination or prophylactic status (not vaccinated, receipt of 1-2 doses, receipt of a booster dose) will be summarized in the demographics table.

5.5.2 Prior drug and non-drug therapies for COVID-19

Prior drug and non-drug therapies for COVID-19 will be summarized, respectively.

5.6 Efficacy Analysis

Unless otherwise specified, the efficacy analysis will be conducted based on FAS, the dataset will be analyzed by the treatment assignment at randomization.

The efficacy endpoints will be listed in the following table.

Table. Efficacy endpoint

Efficacy endpoint	Analytical method	Analysis set	Missing data processing
**Primary endpoint**			
The percentage of participants of VLR after treatment completion within 60 days post-randomization	A Chi-square test will be used (when the total number of the primary endpoint is less than 40 or when the theoretical frequency of a category in a fourfold table is less than 5, or when the *P*-value obtained from the Chi-square test is approximately equal to α, Fisher's exact test will be used)	FAS<br>PPS	Refer to Appendix 1
**Secondary endpoints**			
The percentage of cases in which the viral nucleic acid Ct value decreased by more than 1.5 compared with the day after treatment completion during the follow-up period within 60 days after randomization	The analytical method is the same as the primary endpoint	FAS	Refer to Appendix 1
The percentage of participants who experienced symptom aggregation compared with the day after treatment completion during the follow-up period within 60 days after randomization	The analytical method is the same as the primary endpoint	FAS	Refer to Appendix 1
The percentage of participants who experienced sustained symptom aggregation compared with the day after treatment completion during the follow-up period within 60 days after randomization	The analytical method is the same as the primary endpoint	FAS	Refer to Appendix 1

Time until VLR from completion of treatment	Kaplan-Meier (KM) method and Brookmeyer-Crowley method	FAS	Refer to Appendix 1
The percentage of participants experiencing VLR within 7, 14, 21, and 28 days after treatment completion	The analytical method is the same as the primary endpoint	FAS	Refer to Appendix 1

5.6.1 Primary efficacy endpoint

<u>During the follow-up period within 60 days after randomization, the percentage of participants experiencing VLR after treatment completion.</u>

Viral rebound is defined as an increase in quantified viral load by >0.5 $\log_{10}$ copies/mL in the follow-up period after treatment completion. If a participant does not experience viral rebound, she/he will be censored at the date of the last nucleic acid testing.

The occurrence rate of VLR within 60 days after randomization will be compared using the Chi-square test, unless the total number of primary endpoint is less than 40, or if any theoretical frequency of a category in a fourfold table is less than 5, or if the *P*-value from the Chi-square test is approximately equal to α. In such cases, Fisher's exact test will be used for comparison.

5.6.2 Secondary efficacy endpoints

- Viral load tested by using RT-qPCR for SARS-CoV-2, and a reduction of more than 1.5 Ct values compared to the day after treatment completion. The analytical method is identical to that of the primary analysis for the primary efficacy endpoint.
- Time until VLR from treatment completion

  The Kaplan–Meier method was used to estimate the median time to VLR and Kaplan-Meier survival curves are plotted. The 95% confidence intervals for VLR time are estimated using the Brookmeyer-Crowley method with a log-log transformation to achieve the normality approximation.

- The percentage of participants experiencing VLR within 7, 14, 21, and 28 days after treatment completion

  The analytical method is identical to that of the primary analysis for the primary efficacy endpoint.

- The percentage of participants who experienced symptom aggregation after treatment completion during the follow-up.

  The analytical method is identical to that of the primary analysis for the primary efficacy endpoint.

- The percentage of participants who experienced sustained symptom aggregation after treatment completion during the follow-up.

The analytical method is identical to that of the primary analysis for the primary efficacy endpoint.

5.7 Safety Analysis

Unless otherwise specified, the safety analysis will be performed in SS.

5.7.1 Drug exposure and compliance

The administered doses of JT001 (VV116) and nirmatrelvir–ritonavir will be summarized using descriptive statistics, respectively.

- Actual cumulative dose, defined as the sum of actually taking doses
- Predetermined cumulative dose, defined as the sum of predetermined taking doses
- Compliance, defined as (actual cumulative dose/predetermined cumulative dose) × 100%
- Compliance-based grouping (≥ 80%, < 80%)

5.7.2 Adverse event

All AEs will be coded by MedDRA.

All AE summary tables will only be based on treatment-emergent adverse events (TEAE) (new event or worse than baseline).

The following types of AEs will be summarized by SOC and PT in terms of the number of cases and events.

- TEAE
- TEAEs leading to IMP interruption
- IMP-related TEAEs
- IMP-related serious TEAEs
- IMP-related TEAEs leading to IMP interruption
- IMP-related fatal TEAEs

In addition to the listing of all AEs, listing for SAEs will be presented.

5.7.3 Death

A descriptive summary of death and the primary causes of death will be provided.

5.7.4 Laboratory tests

Descriptive statistics will be used to summarize baseline data of laboratory tests (hematology, clinical biochemistry, and other laboratory examinations, as well as the changes from baseline measurements [if applicable]).

5.8 Subgroup Analysis

The subgroup analysis will be performed on the primary endpoint to evaluate the consistency in intervention effects between the following subgroups.

- Age group: <40 years; 40-60 years; ≥ 60 years
- Gender: women; men
- BMI: ≥25 kg/m^2; < 25 kg/m^2
- Diabetes: with; without
- Cardiovascular diseases (including hypertension): with; without
- SARS-CoV-2 vaccination: unvaccinated; vaccinated (no booster); vaccinated (booster)
- Time from first positive test for SARS-CoV-2 to first dose of study drug: < 3 days; ≥ 3 days

5.9 Interim Analysis

No scheduled interim analysis will be conducted in this study.

6 Appendix 1

(1) The missing nucleic acid results (Ct values) are imputed:

- For participants with missing SARS-CoV-2 test results on D6 after randomization (the day after treatment completion), the Ct value on the most adjacent visit before D15 is assigned to D6.
- For participants with missing SARS-CoV-2 test at visit days after D6, if the test results before and after the missing detection are negative, they are imputed with negative SARS-CoV-2 test results.
- For participants with missing SARS-CoV-2 test results at visit days after D6, if the test results before and after the missing detection are positive, the mean value of the SARS-CoV-2 test results before and after the missing detection is used for imputation.
- For participants with missing SARS-CoV-2 test results at visit days after D6, if the test result before the missing detection is positive and the test result after the missing detection is negative, it is imputed with a negative SARS-CoV-2 test result.
- For participants with missing SARS-CoV-2 test results at visit days after D6, if the test result before the missing detection is negative and the result after the missing detection is positive, it is imputed with a negative SARS-CoV-2 test result.

(2) The Last Observation Carried Forward (LOCF) method is used to impute missing data for COVID-19-related symptom scores after D6, which means that the missing value is imputed with the previously available observation. Missingness on D6 is not imputed and participants are excluded from symptom analysis if they miss symptom score on D6.

(3) No attempt will be made to assign a value to missing viral RNA copy numbers.

(4) If laboratory test values are recorded as being below (and including) or above (and including) the detection range value (e.g., $< x$, $\leq x$, $> x$, $\geq x$), they will be considered as the detection range value (x) in summary descriptive statistics. However, in the data list, they will still be recorded as '< x', '≤ x ', '> x' or '≥ x' as reported in the Case Report Form (CRF).

(5) When calculating age, if one date is missing, the missing date is estimated as follows, unless otherwise specified:

- If the year, month, and day are all missing, it is a missing data.
- If only the year is available without any missing, July 1st is assumed for the missing month and day.
- If only the day is missing, it is assumed to be the 15th of that month.

(6) When the relationship (causality) between an adverse event (AE) and the study drug is missing, the AE will be classified as a drug-related adverse event.

(7) The missing data of safety will not be imputed.
