# Supplementary material for: COVID-19 Rebound After VV116 vs Nirmatrelvir-Ritonavir Treatment: A Randomized Clinical Trial
Source: JAMA Netw Open. 2024 Mar 13;7(3):e241765. doi: 10.1001/jamanetworkopen.2024.1765 (PMC10938176; doi:10.1001/jamanetworkopen.2024.1765)
Supplement: Supplement 2. — eMethods. Genome Sequencing Procedures eReferences. eTable 1. Primary and Secondary Outcomes in the Per Protocol Set eTable 2. Post-Hoc Analysis of Characteristics Associated With Viral Rebound Rate eTable 3. Post-Hoc Analysis of Characteristics Associated With Viral Rebound Rate in VV116 Group eTable 4. Post-Hoc Analysis of Characteristics Associated With Viral Rebound Rate in Nirmatrelvir-Ritonavir Group eTable 5. Adverse Events in the Safety Analysis Set eTable 6. Whole-Genome Sequencing of SARS-CoV-2 in 24 Cases eFigure 1. Enrollment of Study Participants eFigure 2. Median (Interquartile Range) Score for COVID-19–Related Target Symptoms in Patients Receiving VV116 or Nirmatrelvir-Ritonavir by Follow-Up Days eFigure 3. Trajectories of Viral Copy Numbers (log10-Transformed) by Groups of Rebound and Not Rebound eFigure 4. SARS-CoV-2 Variants, Ct Values, and Symptom Scores in 24 Cases [file jamanetwopen-e241765-s002.pdf]

Supplementary Online Content

Yang Z, Xu Y, Zheng R, et al. COVID-19 rebound after VV116 vs nirmatrelvir-ritonavir treatment: a randomized clinical trial. *JAMA Netw Open.* 2024;7(3):e241765. doi:10.1001/jamanetworkopen.2024.1765

eMethods. Genome Sequencing Procedures
eReferences.
eTable 1. Primary and Secondary Outcomes in the Per Protocol Set
eTable 2. Post-Hoc Analysis of Characteristics Associated With Viral Rebound Rate
eTable 3. Post-Hoc Analysis of Characteristics Associated With Viral Rebound Rate in VV116 Group
eTable 4. Post-Hoc Analysis of Characteristics Associated With Viral Rebound Rate in Nirmatrelvir-Ritonavir Group
eTable 5. Adverse Events in the Safety Analysis Set
eTable 6. Whole-Genome Sequencing of SARS-CoV-2 in 24 Cases
eFigure 1. Enrollment of Study Participants
eFigure 2. Median (Interquartile Range) Score for COVID-19–Related Target Symptoms in Patients Receiving VV116 or Nirmatrelvir-Ritonavir by Follow-Up Days
eFigure 3. Trajectories of Viral Copy Numbers (log10-Transformed) by Groups of Rebound and Not Rebound
eFigure 4. SARS-CoV-2 Variants, Ct Values, and Symptom Scores in 24 Cases

This supplementary material has been provided by the authors to give readers additional information about their work.

© 2024 Yang Z et al. *JAMA Network Open.*

eMethods. Genome Sequencing Procedures

RNA extraction and targeting sequencing of SARS-CoV-2: RNA was extracted from swab samples using the Pre-assembled Nucleic Extraction Kit (Magnetic Beads Method) following the manufacturer's instructions (Shanghai BioGerm Medical Technology Co., Ltd, Shanghai, China). The SARS-CoV-2 amplicon library was obtained with the Illumina COVIDSeq ARTIC V4.1 kit following the manufacturer's instructions (Illumina, Inc.). The libraries were sequenced at the MGISEQ 2000AR platform (MGI Tech Co., Ltd, Shenzhen, China) according to PE 150bp protocol. Prior to subsequent analysis, sequencing reads were trimmed using FASTP (version 0.23.2) to remove low-quality regions, adapter sequences, and sequencing primers.

Viral genomic analysis: Sequenced reads were mapped to the reference genome of SARS-CoV-2 (NCBI Accession: NC_045512.2) using BOWTIE2 (v2.4.4),[1] a kmer-based algorithm. In this study, all mapped reads were trimmed and piled up for consensus assembly and variation calls using iVAR (1.3.1).[2] nf-core/viralrecon (v2.5) pipeline was used to coordinate software modules.[3] Lineage analysis was performed by Nextclade (v2.11.0).[4] Bioinformatics analyses were performed on the NRCTM ASTRA supercomputing platform.

© 2024 Yang Z et al. *JAMA Network Open.*

eReferences.

1 Langmead B, Salzberg SL. Fast gapped-read alignment with Bowtie 2. *Nature Methods.* 2012; **9**: 357–359.

2 Grubaugh ND, Gangavarapu K, Quick J, et al. An amplicon-based sequencing framework for accurately measuring intrahost virus diversity using PrimalSeq and iVar. *Genome Biol* 2019; **20**: 8.

3 Ewels PA, Peltzer A, Fillinger S, et al. The nf-core framework for community-curated bioinformatics pipelines. *Nat Biotechnol.* 2020; **38**: 276–278.

4 Aksamentov I, Roemer C, Hodcroft EB, Neher RA. Nextclade: clade assignment, mutation calling and quality control for viral genomes. *Journal of Open Source Software.* 2021; **6**: 3773.

© 2024 Yang Z et al. *JAMA Network Open.*

eTable 1. Primary and Secondary Outcomes in the Per Protocol Set

**Outcomes**	**VV116 (N = 146)**	**Nirmatrelvir–ritonavir (N = 159)**	**p value**
**Primary outcome**			
Viral rebound - no. (%)	29 (19.9%)	37 (23.3%)	0.470
**Secondary outcomes**			
Reduction in Ct value ≥1.5 - no. (%)	34 (22.6%)	33 (21.4%)	0.594
Median time to viral rebound since treatment completion (95% CI) - days	8 (8 - 10)	10 (8 - 10)	0.654
Viral rebound within 7 days after treatment completion – no. (%)	26 (17.8%)	33 (20.8%)	0.515
Viral rebound within 14 days after treatment completion - no. (%)	29 (19.9%)	35 (22.0%)	0.645
Viral rebound within 21 days after treatment completion - no. (%)	29 (19.9%)	36 (22.6%)	0.554
Viral rebound within 28 days after treatment completion - no. (%)	29 (19.9%)	36 (22.6%)	0.554
Symptom aggregation - no. (%)*	36 (24.8%)	33 (21.7%)	0.525
Sustained symptom aggregation - no. (%)*	17 (11.7%)	15 (9.9%)	0.606

* Eight participants were not included in the analysis due to missing symptom score at day 6.

© 2024 Yang Z et al. *JAMA Network Open.*

eTable 2. Post-Hoc Analysis of Characteristics Associated With Viral Rebound Rate

**Characteristics**	**No. of VLR/No. of participants (%)**	**Unadjusted OR (95% CI)**	**Adjusted OR (95% CI)** #
Doses of study drug			
< 5	5/32 (15.6%)	1.00 (Reference)	1.00 (Reference)
5-7	6/22 (27.3%)	1.41 (0.53 – 3.77)	0.98 (0.33 – 2.96)
8-10	61/291 (21.0%)	0.70 (0.26 – 1.89)	0.68 (0.24 – 1.97)
P for trend *	0.672	/	/
Age categories			
Age < 40 years	12/88 (13.6%)	1.00 (Reference)	1.00 (Reference)
Age 40-49 years	12/68 (17.8%)	1.36 (0.57 – 3.24)	1.26 (0.51 – 3.09)
Age 50-59 years	15/72 (20.8%)	1.67 (0.72 – 3.83)	1.91 (0.79 – 4.62)
Age ≥ 60 years	33/117 (28.2%)	**2.49 (1.20 – 5.16)**	**2.64 (1.21 – 5.75)**
P for trend *	**0.009**	/	/
BMI **			
< 25 kg/m^2	46/219 (21.0%)	1.00 (Reference)	1.00 (Reference)
25 - 29.9 kg/m^2	20/92 (21.7%)	1.04 (0.58 – 1.89)	0.96 (0.52 – 1.78)
≥ 30 kg/m^2	2/18 (11.1%)	0.47 (0.10 – 2.12)	0.47 (0.10 – 2.15)
P for trend *	0.570	/	/
Vaccination status **			
Unvaccinated	3/22 (13.6%)	1.00 (Reference)	1.00 (Reference)
1-2 doses	42/191 (22.0%)	1.79 (0.50 – 6.32)	2.03 (0.54 – 7.59)
Boosted dose	27/129 (20.9%)	1.67 (0.46 – 6.09)	1.90 (0.49 – 7.35)
P for trend *	0.739	/	/
Time from first positive test for SARS-CoV-2 to first dose of study drugs			
< 3 days	64 / 280 (22.9%)	1.00 (Reference)	1.00 (Reference)
≥ 3 days	8 / 65 (12.3%)	0.47 (0.22 – 1.05)	0.44 (0.20 – 1.00)
P value	0.059	/	/

* Cochran-Armitage test for trend.
** Missing data from 16 participants for BMI and from 3 participants for vaccination status.
# Odds ratios were mutually adjusted for variables in the table and treatment groups.
BMI, body-mass index; CI, confidence interval; OR, odds ratio; VLR, viral load rebound.

© 2024 Yang Z et al. *JAMA Network Open.*

eTable 3. Post-Hoc Analysis of Characteristics Associated With Viral Rebound Rate in VV116 Group

Characteristics	No. of VLR/No. of participants (%)	Unadjusted OR (95% CI)	Adjusted OR (95% CI) #
Doses of study drug			
< 5	2/13 (15.4%)	1.00 (Reference)	1.00 (Reference)
5-7	3/13 (23.1%)	1.19 (0.31 – 4.61)	1.30 (0.30 – 5.73)
8-10	28/139 (20.1%)	0.72 (0.15 – 3.44)	0.71 (0.13 – 3.87)
P for trend *	0.789	/	/
Age categories			
Age < 40 years	5/32 (15.6%)	1.00 (Reference)	1.00 (Reference)
Age 40-49 years	6/33 (18.2%)	1.64 (0.45 – 5.95)	1.55 (0.39 – 6.17)
Age 50-59 years	8/31 (25.8%)	2.57 (0.75 – 8.83)	2.94 (0.79 – 10.93)
Age ≥ 60 years	14/59 (23.7%)	2.30 (0.76 – 6.98)	2.46 (0.74 – 8.12)
P for trend *	0.121	/	/
BMI **			
< 25 kg/m^2	23/106 (21.7%)	1.00 (Reference)	1.00 (Reference)
25 - 29.9 kg/m^2	7/41 (17.1%)	0.74 (0.29 – 1.89)	0.67 (0.25 – 1.82)
≥ 30 kg/m^2	1/10 (10.0%)	0.40 (0.05 – 3.33)	0.40 (0.05 – 3.53)
P for trend *	0.312	/	/
Vaccination status **			
Unvaccinated	2/12 (16.7%)	1.00 (Reference)	1.00 (Reference)
1-2 doses	16/91 (17.6%)	1.07 (0.21 – 5.34)	1.19 (0.22 – 6.56)
Boosted dose	15/61 (24.6%)	1.63 (0.32 – 8.29)	1.97 (0.35 – 11.10)
P for trend *	0.305	/	/
Time from first positive test for SARS-CoV-2 to first dose of study drugs			
< 3 days	29/134 (21.6%)	1.00 (Reference)	1.00 (Reference)
≥ 3 days	4/31 (12.9%)	0.54 (0.17 – 1.66)	0.58 (0.18 – 1.89)
P value	0.273	/	/

***** Cochran-Armitage test for trend.
** Missing data from 16 participants for BMI and from 3 participants for vaccination status.
# Odds ratios were mutually adjusted for variables in the table.
BMI, body-mass index; CI, confidence interval; OR, odds ratio; VLR, viral load rebound.

© 2024 Yang Z et al. *JAMA Network Open.*

eTable 4. Post-Hoc Analysis of Characteristics Associated With Viral Rebound Rate in Nirmatrelvir-Ritonavir Group

Characteristics	No. of VLR/No. of participants (%)	Unadjusted OR (95% CI)	Adjusted OR (95% CI) #
Doses of study drug			
< 5	3/19 (15.8%)	1.00 (Reference)	1.00 (Reference)
5-7	3/9 (33.3%)	1.80 (0.43 – 7.60)	0.90 (0.15 – 5.36)
8-10	33/152 (21.7%)	0.68 (0.19 – 2.46)	0.76 (0.19 – 3.07)
P for trend *	0.736	/	/
Age categories			
Age < 40 years	7/46 (15.2%)	1.00 (Reference)	1.00 (Reference)
Age 40-49 years	6/35 (17.1%)	1.15 (0.35 – 3.80)	1.08 (0.32 – 3.64)
Age 50-59 years	7/41 (17.1%)	1.15 (0.37 – 3.60)	1.20 (0.34 – 4.16)
Age ≥ 60 years	19/58 (32.8%)	**2.71 (1.03 – 7.19)**	**3.00 (1.05 – 8.58)**
P for trend *	**0.032**	/	/
BMI **			
< 25 kg/m^2	23/113 (20.4%)	1.00 (Reference)	1.00 (Reference)
25 - 29.9 kg/m^2	13/51 (25.5%)	1.34 (0.61 – 2.92)	1.36 (0.58 – 3.16)
≥ 30 kg/m^2	1/8 (12.5%)	0.56 (0.07 – 4.77)	0.64 (0.07 – 5.74)
P for trend *	0.850	/	/
Vaccination status **			
Unvaccinated	1/10 (10.0%)	1.00 (Reference)	1.00 (Reference)
1-2 doses	26/100 (26.0%)	3.16 (0.38 – 26.18)	3.78 (0.42 – 34.29)
Boosted dose	12/68 (17.6%)	1.93 (0.22 – 16.69)	2.19 (0.23 – 20.87)
P for trend *	0.591	/	/
Time from first positive test for SARS-CoV-2 to first dose of study drugs			
< 3 days	35/146 (24.0%)	1.00 (Reference)	1.00 (Reference)
≥ 3 days	4/34 (11.8%)	0.42 (0.14 – 1.28)	0.37 (0.12 – 1.20)
P value	0.120	/	/

***** Cochran-Armitage test for trend.
** Missing data from 16 participants for BMI and from 3 participants for vaccination status.
# Odds ratios were mutually adjusted for variables in the table.
BMI, body-mass index; CI, confidence interval; OR, odds ratio; VLR, viral load rebound.

eTable 5. Adverse Events in the Safety Analysis Set

**Adverse events**	**VV116 (N = 166)**	**Nirmatrelvir–ritonavir (N = 187)**	**p value**
AE leading to discontinuation of study drugs	2 (1.2%)	6 (3.2%)	0.291*
Serious adverse events	4 (2.4%)	6 (3.2%)	0.755*
Hospitalization due to pneumonia	3 (1.8%)	5 (2.7%)	0.727*
Hospitalization due to pulmonary embolism	0 (0%)	1 (0.5%)	/
Hospitalization and death due to pneumonia	1 (0.6%)	0 (0%)	/
SAEs considered by the investigator to be related to the assigned treatment	0 (0%)	0 (0%)	/

* Fisher's exact test was used.
AE, adverse event; SAE, serious adverse event.

© 2024 Yang Z et al. *JAMA Network Open.*

eTable 6. Whole-Genome Sequencing of SARS-CoV-2 in 24 Cases

No.	Visit Day	Input ReadsNumber	Map&Trimmed readsNumber	medianDepth	# SNPs	# INDELs	Nextclade clade	Nextclade_pango	fullName	QC.genomeCoverage	QC.overall Status	QC.missing Data.status	QC. totalMissing
1	D0	31855792	14000445	25796	79	7	22B (Omicron)	BF.7.14	BA.5.2.1.7.14	0.981139016	mediocre	good	564
	D56	61051266	15985189	17760	78	6	22B (Omicron)	BF.7.14	BA.5.2.1.7.14	0.981540314	mediocre	good	552
2	D0	16342792	12720541	51003	78	5	22B (Omicron)	DY.1	BA.5.2.48.1	0.997291242	good	good	81
	D8	15811318	11317480	43860	76	5	22B (Omicron)	DY.1	BA.5.2.48.1	0.997592215	good	good	72
3	D0	15305316	8357486	25208	81	5	22B (Omicron)	BA.5.2.48	BA.5.2.48	0.986790623	good	good	395
	D8	13086106	4514394	9581	94	5	22B (Omicron)	BA.5.2.48	BA.5.2.48	0.990301976	good	good	290
4	D0	13270804	8024455	28330	80	6	22B (Omicron)	BA.5.2.48	BA.5.2.48	0.997558773	good	good	73
	D10	14774246	8276812	29738	79	5	22B (Omicron)	BA.5.2.48	BA.5.2.48	0.997458449	good	good	76
5	D0	14660420	11356336	43192	75	5	22B (Omicron)	BF.7.14	BA.5.2.1.7.14	0.996889944	good	good	93
	D12	17787846	13834385	55731	76	5	22B (Omicron)	BF.7.14	BA.5.2.1.7.14	0.997525332	good	good	74
6	D0	12175772	7404962	22426	79	5	22B (Omicron)	BF.7.14	BA.5.2.1.7.14	0.985620172	mediocre	good	430
	D14	14286366	6499887	14062	81	11	22B (Omicron)	BF.7.14	BA.5.2.1.7.14	0.925559308	mediocre	mediocre	2226
7	D0	12296180	8291514	31728	87	4	22B (Omicron)	BA.5.2	BA.5.2	0.996254556	good	good	112
	D10	13425728	7821073	24708	85	4	22B (Omicron)	BA.5.2	BA.5.2	0.995518844	good	good	134
8	D0	13957866	10452467	41179	76	5	22B (Omicron)	BF.7.14	BA.5.2.1.7.14	0.992542554	mediocre	good	223
	D8	16038350	10289130	37377	75	5	22B (Omicron)	BF.7.14	BA.5.2.1.7.14	0.996020466	mediocre	good	119
9	D0	14120836	5794542	15000	79	5	22B (Omicron)	BA.5.2.48	BA.5.2.48	0.974751697	good	good	755
	D8	10369112	5946656	12257	85	7	22B (Omicron)	BA.5.2.48	BA.5.2.48	0.934254088	mediocre	mediocre	1966
10	D0	17277912	12368425	47837	75	5	22B (Omicron)	BF.7.14	BA.5.2.1.7.14	0.997692539	good	good	69
	D10	16740982	10166771	39973	75	5	22B (Omicron)	BF.7.14	BA.5.2.1.7.14	0.997625656	mediocre	good	71
11	D0	19083484	14700246	54668	78	5	22B (Omicron)	BF.7.14	BA.5.2.1.7.14	0.997558773	good	good	73
	D8	7716306	5157450	17855	76	5	22B (Omicron)	BF.7.14	BA.5.2.1.7.14	0.982376350	good	good	527
12	D0	19961062	15468947	60175	75	5	22B (Omicron)	BA.5.2.48	BA.5.2.48	0.996020466	good	good	119

© 2024 Yang Z et al. *JAMA Network Open.*

	D8	15205132	9803131	37670	76	5	22B (Omicron)	BA.5.2.48	BA.5.2.48	0.995987025	good	good	120
13	D0	15536844	11490699	43787	77	5	22B (Omicron)	BF.7.14	BA.5.2.1.7.14	0.997592215	mediocre	good	72
	D8	16068514	11793022	44025	77	5	22B (Omicron)	BF.7.14	BA.5.2.1.7.14	0.997558773	good	good	73
14	D0	13579524	8534615	30344	87	5	22B (Omicron)	BA.5.2.48	BA.5.2.48	0.995987025	good	good	120
	D10	14346304	10185351	36713	84	5	22B (Omicron)	BA.5.2.48	BA.5.2.48	0.995987025	good	good	120
15	D0	17464250	11972896	47189	76	5	22B (Omicron)	BF.7.14	BA.5.2.1.7.14	0.996254556	mediocre	good	112
	D8	12638434	9202896	36385	77	5	22B (Omicron)	BF.7.14	BA.5.2.1.7.14	0.997926629	good	good	62
16	D0	16068932	11301969	44393	80	5	22B (Omicron)	BA.5.2.50	BA.5.2.50	0.997525332	good	good	74
	D10	15385784	11135724	42311	80	5	22B (Omicron)	BA.5.2.50	BA.5.2.50	0.997190917	good	good	84
17	D0	11062114	8061335	30153	79	5	22B (Omicron)	DY.1	BA.5.2.48.1	0.995987025	good	good	120
	D8	11995450	7342031	25955	77	5	22B (Omicron)	DY.1	BA.5.2.48.1	0.986021469	good	good	418
18	D0	7433278	4784006	18245	76	6	22B (Omicron)	DY.1	BA.5.2.48.1	0.995987025	good	good	120
	D10	16348026	11800658	41887	79	6	22B (Omicron)	DY.1	BA.5.2.48.1	0.995987025	good	good	120
19	D0	17360822	12974962	51691	77	5	22B (Omicron)	BA.5.2.48	BA.5.2.48	0.997558773	good	good	73
	D8	12236636	9324772	36826	76	5	22B (Omicron)	BA.5.2.48	BA.5.2.48	0.997725981	good	good	68
20	D0	15523728	11037980	42123	75	5	22B (Omicron)	BF.7.14	BA.5.2.1.7.14	0.997859747	mediocre	good	64
	D8	17249408	11946432	45470	75	5	22B (Omicron)	BF.7.14	BA.5.2.1.7.14	0.996254556	mediocre	good	112
21	D0	17205108	13335392	49990	77	5	22B (Omicron)	BA.5.2.48	BA.5.2.48	0.997592215	good	good	72
	D16	13155656	9754024	36568	76	5	22B (Omicron)	BA.5.2.48	BA.5.2.48	0.997625656	good	good	71
22	D0	19532768	14040370	51141	76	6	22B (Omicron)	BF.7.14	BA.5.2.1.7.14	0.997625656	good	good	71
	D12	8079472	6108908	21967	78	6	22B (Omicron)	BF.7.14	BA.5.2.1.7.14	0.992542554	good	good	223
23	D0	20355556	15278583	57637	77	6	22B (Omicron)	BA.5.2.48	BA.5.2.48	0.997792864	good	good	66
	D8	16689682	12500396	47018	78	6	22B (Omicron)	BA.5.2.48	BA.5.2.48	0.997592215	good	good	72
24	D0	13156890	6925313	21832	78	5	22B (Omicron)	BA.5.2.48	BA.5.2.48	0.975320202	good	good	738
	D10	11659676	7254342	20544	80	5	22B (Omicron)	BA.5.2.48	BA.5.2.48	0.968665351	mediocre	good	937

© 2024 Yang Z et al. *JAMA Network Open.*

eFigure 1. Enrollment of Study Participants

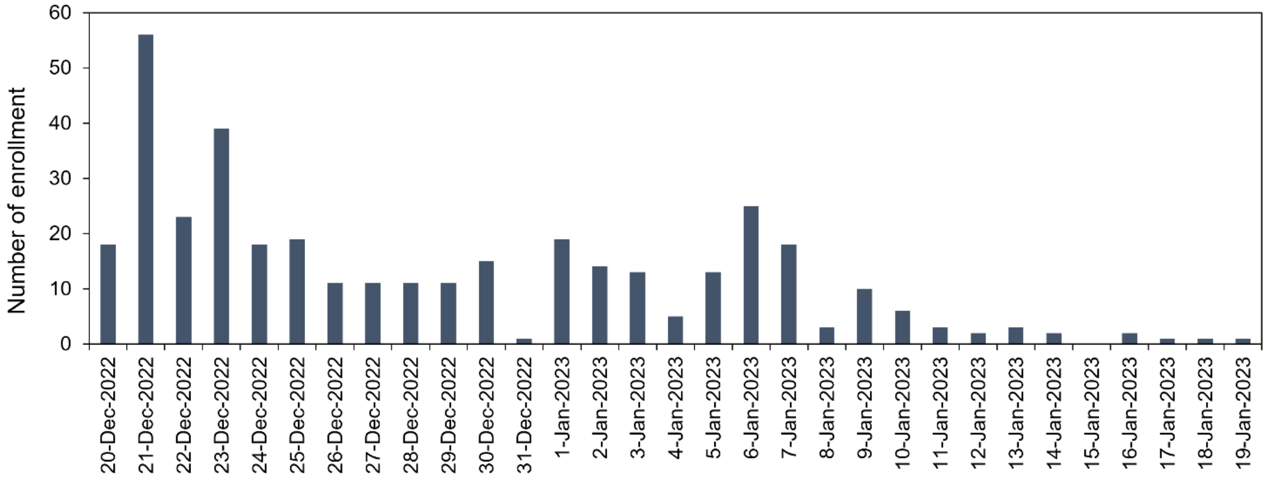

© 2024 Yang Z et al. *JAMA Network Open.*

eFigure 2. Median (Interquartile Range) Score for COVID-19–Related Target Symptoms in Patients Receiving VV116 or Nirmatrelvir-Ritonavir by Follow-Up Days

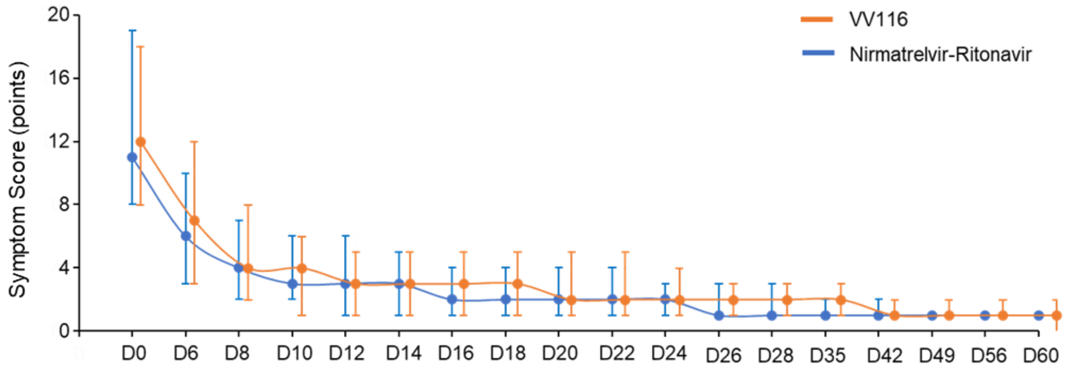

© 2024 Yang Z et al. *JAMA Network Open.*

eFigure 3. Trajectories of Viral Copy Numbers (log10-Transformed) by Groups of Rebound and Not Rebound

Generalized additive mixed models were used to fit the trajectories of log-transformed viral RNA copy numbers per mL plus 1 (because many data points are 0) by groups of rebound and not rebound. Solid lines represent smoothed fitted viral copy numbers (log10-transformed) and grey areas represent point-related 95% confidence intervals.

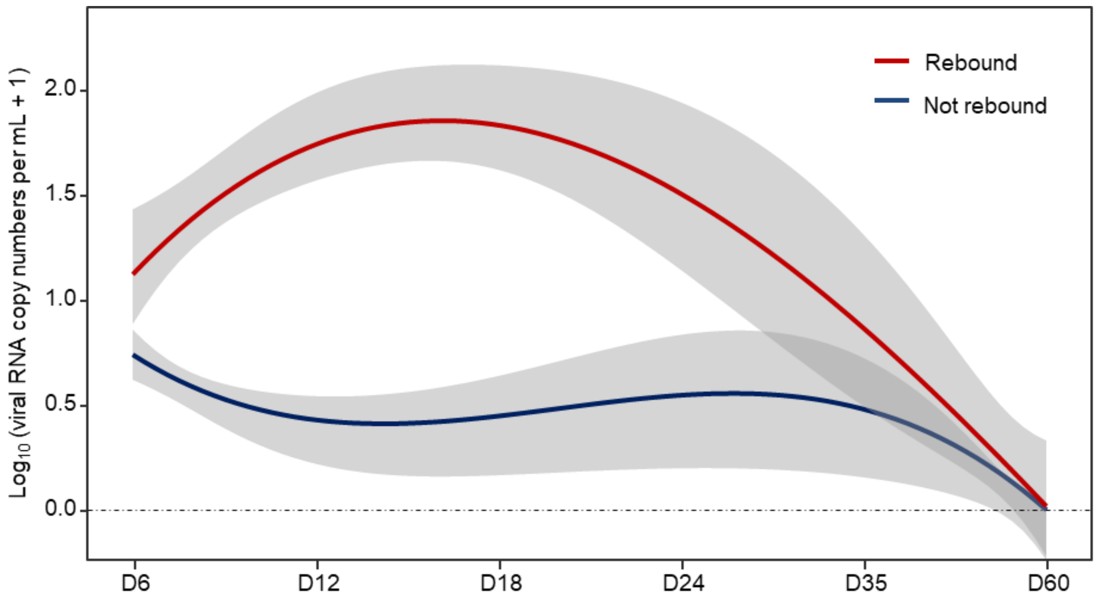

© 2024 Yang Z et al. *JAMA Network Open.*

eFigure 4. SARS-CoV-2 Variants, Ct Values, and Symptom Scores in 24 Cases

No.	Test	D0	D6	D8	D10	D12	D14	D16	D18	D20	D22	D56
	Virant	BF.7										BF.7
1	Ct Value	30·20	37·23	(-)	(-)	(-)	(-)	(-)	(-)	(-)	(-)	32·74
	Symptom Score (points)	9	8	6	5	6	3	1	1	1	1	1
	Virant	BA.5.2		BA.5.2								
2	Ct Value	25·07	(-)	34·96	(-)	(-)	(-)	(-)	(-)	(-)	(-)	(-)
	Symptom Score (points)	6	1	0	0	1	0	0	0	0	0	0
	Virant	BA.5.2		BA.5.2								
3	Ct Value	29·19	(-)	34·57	36·63	(-)	(-)	(-)	(-)	(-)	(-)	(-)
	Symptom Score (points)	8	4	5	3	6	5	3	1	2	3	1
	Virant	BA.5.2			BA.5.2							
4	Ct Value	27·61	(-)	(-)	31·64	29·27	27·80	(-)	(-)	(-)	(-)	(-)
	Symptom Score (points)	6	14	6	12	10	9	10	9	9	9	5
	Virant	BF.7				BF.7						
5	Ct Value	24·00	34·98	33·80	32·62	31·44	(-)	(-)	(-)	(-)	(-)	(-)
	Symptom Score (points)	24	26	14	10	4	3	6	3	3	2	2
	Virant	BF.7					BF.7					
6	Ct Value	27·96	(-)	(-)	(-)	(-)	33·79	(-)	33·13	35·64	(-)	(-)
	Symptom Score (points)	25	14	23	19	13	16	15	10	14	9	9
	Virant	BA.5.2			BA.5.2							
7	Ct Value	27·29	(-)	(-)	31·39	25·20	29·43	29·18	(-)	(-)	(-)	(-)
	Symptom Score (points)	13	10	7	7	8	8	5	8	6	5	0
	Virant	BF.7		BF.7								
8	Ct Value	26·77	(-)	31·42	(-)	(-)	(-)	(-)	(-)	(-)	(-)	(-)
	Symptom Score (points)	17	8	6	4	5	3	3	3	3	1	2
	Virant	BA.5.2		BA.5.2								
9	Ct Value	32·86	29·19	23·71	34·04	(-)	31·24	(-)	(-)	(-)	(-)	(-)
	Symptom Score (points)	14	7	3	2	2	2	2	1	1	1	1
	Virant	BF.7			BF.7							
10	Ct Value	27·26	33·92	(-)	23·19	37·38	38·02	(-)	(-)	(-)	(-)	(-)
	Symptom Score (points)	22	12	5	7	9	5	5	6	5	5	1
	Virant	BF.7		BF.7								
11	Ct Value	25·49	35·68	34·19	29·37	36·89	36·70	(-)	(-)	(-)	(-)	(-)
	Symptom Score (points)	11	33	6	0	2	1	1	1	1	4	1
	Virant	BA.5.2		BA.5.2								
12	Ct Value	24·02	(-)	22·47	(-)	(-)	(-)	(-)	(-)	(-)	(-)	(-)
	Symptom Score (points)	7	9	9	9	9	9	9	9	9	9	9
	Virant	BF.7		BF.7								
13	Ct Value	22·94	34·63	28·12	(-)	(-)	(-)	(-)	(-)	(-)	(-)	(-)
	Symptom Score (points)	14	26	7	8	8	3	4	3	2	3	2
	Virant	BA.5.2			BA.5.2							
14	Ct Value	30·12	(-)	(-)	32·01	(-)	(-)	(-)	(-)	(-)	(-)	(-)
	Symptom Score (points)	19	13	13	13	13	3	7	3	7	7	1
	Virant	BF.7		BF.7								
15	Ct Value	31·73	26·41	18·91	32·00	33·11	31·70	(-)	35·53	(-)	(-)	(-)
	Symptom Score (points)	3	44	17	8	3	11	3	8	10	8	2
	Virant	BA.5.2			BA.5.2							
16	Ct Value	23·96	(-)	(-)	33·22	(-)	(-)	(-)	(-)	(-)	(-)	(-)
	Symptom Score (points)	20	6	1	9	8	5	5	9	6	5	1
	Virant	BA.5.2		BA.5.2								
17	Ct Value	27·11	(-)	27·27	31·00	(-)	31·47	21·19	(-)	(-)	(-)	(-)
	Symptom Score (points)	15	1	1	1	5	2	2	0	0	0	0
	Virant	BA.5.2			BA.5.2							
18	Ct Value	29·07	(-)	(-)	34·42	(-)	(-)	(-)	(-)	(-)	(-)	(-)
	Symptom Score (points)	13	6	5	5	7	6	3	3	3	3	1
	Virant	BA.5.2		BA.5.2								
19	Ct Value	24·11	36·58	33·64	(-)	(-)	(-)	(-)	(-)	(-)	(-)	(-)
	Symptom Score (points)	9	2	2	4	2	2	2	0	0	1	1
	Virant	BF.7		BF.7								
20	Ct Value	24·54	29·79	27·02	34·41	25·46	27·04	26·27	35·05	27·66	(-)	(-)
	Symptom Score (points)	0	17	18	10	11	11	7	11	8	6	1
	Virant	BA.5.2						BA.5.2				
21	Ct Value	24·38	36·64	(-)	36·65	(-)	(-)	34·18	(-)	(-)	(-)	(-)
	Symptom Score (points)	17	0	3	3	0	2	0	0	0	0	0
	Virant	BF.7				BF.7						
22	Ct Value	23·32	38·75	(-)	(-)	29·80	34·66	39·49	(-)	(-)	35·20	(-)
	Symptom Score (points)	10	8	3	3	1	0	0	1	0	1	1
	Virant	BA.5.2		BA.5.2								
23	Ct Value	22·76	(-)	33·40	33·68	(-)	(-)	(-)	(-)	(-)	(-)	(-)
	Symptom Score (points)	11	3	3	3	3	1	0	0	0	0	0
	Virant	BA.5.2			BA.5.2							
24	Ct Value	29·00	(-)	(-)	29·86	(-)	(-)	(-)	(-)	(-)	(-)	(-)
	Symptom Score (points)	15	10	10	11	9	9	7	6	2	5	1

SARS-CoV-2 variant
Reduction in Ct value >=1.5
Symptom aggregation
No progression
Imputed data

© 2024 Yang Z et al. *JAMA Network Open.*
