# Supplementary material for: COVID-19 Rebound After VV116 vs Nirmatrelvir-Ritonavir Treatment: A Randomized Clinical Trial
Source: JAMA Netw Open. 2024 Mar 13;7(3):e241765. doi: 10.1001/jamanetworkopen.2024.1765 (PMC10938176; doi:10.1001/jamanetworkopen.2024.1765)
Supplement: Supplement 3. — Data Sharing Statement [file jamanetwopen-e241765-s003.pdf]

Data Sharing Statement

Yang. COVID-19 Rebound After VV116 vs Nirmatrelvir-Ritonavir Treatment. *JAMA Netw Open*. Published March 13, 2024. doi:10.1001/jamanetworkopen.2024.1765

Data
Data available: No

Additional Information
Explanation for why data not available: Individual patient data will not be available to others as per the policy of Medical Ethics Committee of Ruijin Hospital.
